# Decreased Enterobacteriaceae translocation due to gut microbiota remodeling mediates the alleviation of premature aging by a high‐fat diet

**DOI:** 10.1111/acel.13760

**Published:** 2022-12-25

**Authors:** Kang Xu, Yannan Guo, Yida Wang, Yu Ren, Vivien Low, Sungyun Cho, Lu Ping, Kezheng Peng, Xue Li, Ying Qiu, Qingfei Liu, Zhongchi Li, Zhao Wang

**Affiliations:** ^1^ Protein Science Key Laboratory of the Ministry of Education School of Pharmaceutical Sciences Tsinghua University Beijing China; ^2^ School of Basic Medical Sciences Capital Medical University Beijing China; ^3^ Department of Pharmacology Weill Cornell Medicine New York New York USA; ^4^ Peking Union Medical College Beijing China; ^5^ School of Medicine Tsinghua University Beijing China; ^6^ Lead Contract

**Keywords:** Enterobacteriaceae translocation, Gut dysbiosis, high‐fat diet, premature aging, SIRT6

## Abstract

Aging‐associated microbial dysbiosis exacerbates various disorders and dysfunctions, and is a major contributor to morbidity and mortality in the elderly, but the underlying cause of this aging‐related syndrome is confusing. SIRT6 knockout (SIRT6 KO) mice undergo premature aging and succumb to death by 4 weeks, and are therefore useful as a premature aging research model. Here, fecal microbiota transplantation from SIRT6 KO mice into wild‐type (WT) mice phenocopies the gut dysbiosis and premature aging observed in SIRT6 KO mice. Conversely, an expanded lifespan was observed in SIRT6 KO mice when transplanted with microbiota from WT mice. Antibiotic cocktail treatment attenuated inflammation and cell senescence in KO mice, directly suggesting that gut dysbiosis contributes to the premature aging of SIRT6 KO mice. Increased Enterobacteriaceae translocation, driven by the overgrowth of *Escherichia coli*, is the likely mechanism for the premature aging effects of microbiome dysregulation, which could be reversed by a high‐fat diet. Our results provide a mechanism for the causal link between gut dysbiosis and aging, and support a beneficial effect of a high‐fat diet for correcting gut dysbiosis and alleviating premature aging. This study provides a rationale for the integration of microbiome‐based high‐fat diets into therapeutic interventions against aging‐associated diseases.

## Graphical abstract

SIRT6 KO mice are characterized by a gut dysbiosis, particularly due to overabundant *Escherichia coli* colonization in gut and Enterobacteriaceae translocation into visceral organs, partly resulting in a shortened lifespan and premature aging. Crucially, a high‐fat diet improves gut dysbiosis, which positively correlates with extended lifespan and rescues premature aging in SIRT6 KO mice.

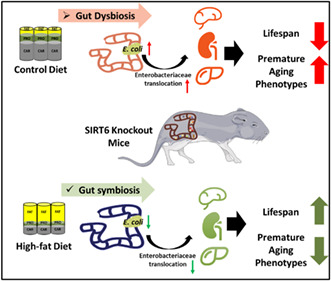

## 1 INTRODUCTION

Increasing evidence demonstrates that gut microbiota dysregulation lies at the core of many aging‐related changes, including immune dysfunction and increased susceptibility to diseases (Badal et al., 2020; DeJong et al., 2020). Gut physiology changes with age and these changes likely account for the persistence or outgrowth of certain bacteria over others (Amato et al., 2019). For example, the production of mucin, a nutrient source for Akkermansiaceae, reduces with age, resulting in the loss of these bacteria in the elderly (Elderman et al., 2017) Erysipelotrichaceae increases due to its ability to adapt to aging‐related changes in the local redox and nutrient environments (Chen et al., 2017). The resulting gut dysbiosis aggravates degenerative pathologies and unhealthy aging (Kim & Jazwinski, 2018). At present, increased systemic inflammation and gut permeability are recognized to result from gut dysbiosis during aging (Falk et al., 1998; Thevaranjan et al., 2017). However, it has not been determined whether this is due to the overgrowth of specific species, reduction of functional microbiota, changes in microbe‐microbe interactions, or other reasons (Thevaranjan et al., 2017). Therefore, the mechanism of gut dysbiosis in aging is still to be clarified.

SIRT6 knockout (SIRT6 KO) mice develop acute loss of subcutaneous fat, spontaneous colitis, hypoglycemia, osteopenia, and muscle atrophy at approximately 3 weeks of age, succumbing to death by 4 weeks; therefore, they are useful as a premature aging research model (Li et al., 2020; Mostoslavsky et al., 2006). In this study, we used SIRT6 KO mice to functionally assess how changes in the gut microbiota contribute to aging and whether the gut microbiota can be targeted to improve aging phenotypes.

The remodeling of the gut microbiome by dietary intervention affects whole‐body metabolism, nutrient homeostasis, and immunity (Yang et al., 2017). For instance, functional gut microbiome remodeling contributes to caloric restriction‐induced metabolic improvements and lifespan extension (Fabbiano et al., 2018; Zhang et al., 2013). This has led us to speculate that the gut microbiome might mediate some of the metabolic effects of other diets. The ketogenic diet, characterized by little‐to‐no carbohydrate and high‐fat consumption, has been reported to extend longevity, preserve motor function, improve memory, and reduce mortality in aging mice (Newman et al., 2017; Roberts et al., 2018). A high‐fat diet can rescue premature aging, including the metabolic and cerebellar phenotypes in Cockayne syndrome, through the activation of Sirt1 (Scheibye‐Knudsen et al., 2014). Moreover, an isocaloric moderately high‐fat diet could prolong the lifespan of rats and flies (Shi et al., 2021). Despite these correlations, there is a dearth of information on how the microbiome is altered and the contribution of these changes to phenotypical aging improvements by such diets. According to a recent study, a ketogenic diet alters the gut microbiome and, subsequently, the immune response, resulting in decreased intestinal pro‐inflammatory Th17 cells (Ang et al., 2020). Additionally, our previous studies have shown that high‐fat diets in SIRT6 KO mice can extend their lifespan and reverse metabolic disorders (Li et al., 2020). We hypothesized that a high‐fat diet might rebalance gut microbiota dysbiosis, resulting in the amelioration of premature aging phenotypes.

In the current study, we described that increased Enterobacteriaceae translocation to visceral organs driven by the overgrowth of *Escherichia coli* contributes to the premature aging phenotype in SIRT6 KO mice. Intriguingly, a high‐fat diet rebalances gut dysbiosis, which mediates health span and lifespan extension in SIRT6 KO mice. Our study provides a theoretical foundation for microbiome‐based dietary interventions for aging and age‐related diseases.

## 2 METHODS

### 2.1 Mouse generation and study

SIRT6^tm1.1Cxd^ mice of a 129S6/SvEvTac (129Sv) background were purchased from the Jackson Laboratory. Male 129Sv SIRT6^tm1.1Cxd^ mice were mated with C57BL/6J CMV‐Cre female mice (Nanjing Biomedical Research Institute) to generate F1 heterozygous mice. F1 heterozygous mice repeatedly backcrossed to 129Sv wild‐type (WT) mice for more than 10 generations, 99.9% of its genetic composition will be the genetic background of 129Sv, and then, 129Sv SIRT6 heterozygous (SIRT6+/−) mice were interbred to generate WT, SIRT6 KO, and SIRT6+/− mice. The mice were housed at the Laboratory Animal Research Center, Tsinghua University. All mouse experiments were performed on males, complied with regulations and ethics guidelines, and were approved by the International Animal Care and Use Committee of Tsinghua University. Heterozygous breeding produced progeny with the expected Mendelian ratios, and heterozygous mice were used as breeders for our studies. Animal rooms were maintained at 23°C with a 12‐h light/dark cycle. Mice were identified by genotyping. After tail or toe clipping, tissues were digested for 3 h at 55°C in 500 μl of 1 M Tris–HCl, 5 M NaCl, 0.5 M EDTA, and 10% SDS. Protease K and RNase A were also added to the lysis buffer. Isopropanol and ethyl alcohol were used for DNA extraction. Then, the following primers were used for PCR: forward: 5′‐AGTGAGGGGCTAATGGGAAC‐3′; reverse: 5′‐CTGACGGTGTCTTCACAAACTCAC‐3′. If the PCR product was 600 bp long, we considered the genotype to be SIRT6 knockout whereas 2000 bp indicated WT.

SIRT6 knockout (SIRT6 KO, 129Sv) mice, wild type (WT, 129Sv), and SIRT6 heterozygous (SIRT6+/−, 129Sv) littermates were individually caged after weaning with a plaything to allow for acclimation to the animal facility. Fecal samples were obtained under sterile conditions and stored in a sterile 1.5 ml centrifuge tube at −80°C until microbiome profiling analysis. Mice were sacrificed with carbon dioxide, and blood collection was performed immediately via cardiac puncture. Serum was obtained after centrifugation at 1500 *g* for 15 min at 4°C and was frozen at −80°C until thawing for an assay. Then, the colon, intestine, and other tissues were removed and divided separately. Tissues prepared for section staining were post‐fixed in phosphate‐buffered 4% paraformaldehyde, maintained at pH 7.4, and stored at 4°C, whereas tissues prepared for RT‐qPCR and Western blots were snap frozen and stored at −80°C for further analysis.

### 2.2 Antibiotic treatment

Nineteen‐day‐old (after weaning) KO mice were orally given for 3 consecutive days with 100 μl phosphate‐buffered saline (PBS; KO + PBS) or antibiotic cocktail that contained 1 g/L ampicillin, 1 g/L neomycin, 1 g/L metronidazole, and 0.5 g/L vancomycin hydrochloride (KO + Antibiotic). All the mice were singly housed after weaning with a plaything to allow for acclimation to the animal facility. Feces and tissues were collected when mice were 28 days old.

### 2.3 High‐fat diet feeding

Three‐week‐old SIRT6‐knockout (129Sv) and WT mice (129Sv) were fed a control standard AIN‐93G diet (abbreviated as CD) containing 64% carbohydrates, 19% protein, and 17% fat or a high‐fat diet (abbreviated as HD) consisting of AIN‐93G with 65% of calories from fat, principally hydrogenated coconut oil (Table S2). The high‐fat diet contained 16% carbohydrates, 19% protein, and 65% fat. The food supplied to the SIRT6 knockout mice should be accessible, and thus, we placed it, control or high‐fat food, on the bedding considering the weakness and smaller body size of SIRT6 KO mice. In addition, the water bottle was also specially made to ensure the accessibility of water for KO mice. The fiber and moisture content were the same in both the control diet and the high‐fat diet, which were 5% (w/w) and 8% (w/w), respectively. The amounts of the high‐fat and control diets were calculated as caloric intake per day per body weight with 4‐week‐old KO or WT mice. The dietary intervention lasted for 1 week. Feces collection and dissection were carried out after 1 week of high‐fat diet feeding. For the calculation of the survival rate, mice were raised to the end of the fifth week.

### 2.4 Fecal microbiota transplantation

Three‐month‐old C57BL/6J male mice were used as recipients for microbiota transplantation. WT‐WT (WT mice transplanted with fecal microbiota from WT mice), KO‐WT (WT mice transplanted with fecal microbiota from SIRT6 KO mice), KOHD‐WT (WT mice transplanted with fecal microbiota from KO mice who were fed with a high‐fat diet). Before transplantation, mice were treated for 4 consecutive weeks with an antibiotic cocktail in drinking water that contained 1 g/L ampicillin (Sigma), 1 g/L neomycin (Sigma), 1 g/L metronidazole (Sigma), and 0.5 g/L vancomycin hydrochloride (BIORIGIN). The drinking solution was renewed every 2 days. Then, mice were orally given 200 μl of the microbiota suspension every other day for 4 weeks (with each daily dose being administered by oral gavage after 2 h fast), starting the first day after the antibiotic cycle. Feces for further analysis were collected 72 h after fecal microbiota transplantation (FMT). For the microbiota suspension preparation, 300 mg fecal pellets were resuspended with a vortex in 1 ml PBS and then centrifuged at 1500 *g* for 5 min to remove insolubilized material, and the supernatant was stored at −80°C in a freezer. Feces collection and animal dissection were carried out after the microbiota transplantation. Body weight and tissue weight were recorded. Tissue samples were kept at 4°C or −80°C in a freezer, separately. Three to five fecal pellets of each mouse were randomly selected, and the diameter of each fecal pellet was measured using a vernier caliper. The average fecal diameter of each mouse was calculated and then presented in a histogram.

FMT from WT or SIRT6 KO donor mice started when SIRT6 KO recipient mice were 19 days old (after weaning) without antibiotic treatment (Stebegg et al., 2019; WT‐KO: SIRT6 KO mice transplanted with fecal microbiota from WT mice; KO‐KO: SIRT6 KO mice transplanted with fecal microbiota from SIRT6 KO mice). Feces from WT littermates or KO mice were collected just before the FMT and microbiota suspension was prepared as previously mentioned. Microbiota suspension was carefully administered orally to mice. SIRT6 KO mice were given microbiota suspension of 200 μl per day for 1 week. After 1 week, mice received the microbiota suspension twice a week until natural death and the lifespan of mice was recorded. During FMT, cages of recipient mice (SIRT6 KO) were replenished with dirty bedding and fecal pellets from WT or KO mice three times a week. Understandably, the KO mice could obtain the gut microbiota from WT or KO as much as possible because of their coprophagy. Feces and tissues were collected when mice were 28 days old.

FMT from KOHD donor mice started when SIRT6 KO recipients were 19 days old (after weaning) without antibiotic treatment (KOHD‐KO: SIRT6 KO mice transplanted with fecal microbiota from KOHD mice). Feces from KOHD mice were collected just before the FMT, and microbiota suspension was prepared as previously mentioned. Microbiota suspension was carefully administered orally to mice. SIRT6 KO mice were given microbiota suspension of 200 μl per day for 1 week. During FMT, cages of recipient mice (SIRT6 KO) were replenished with dirty bedding and fecal pellets from KOHD mice three times a week. Understandably, the KO mice could obtain the gut microbiota from KOHD as much as possible because of their coprophagy. Feces and tissues were collected when mice were 28 days old.

### 2.5 *Escherichia coli* culture and oral supplementation

Feces from SIRT6 knockout mice were resuspended in Tryptic Soy Broth and then incubated at 37°C to the mid‐exponential phase. The cultures were centrifuged at 6700 *g* for 5 min, and the bacterial pellets were washed twice in PBS. The bacterial suspension was then diluted in 100‐fold serial dilutions and plated on *E. coli* chromogenic medium (CHROM; Solarbio, LA0780). CHROM plates were incubated in the dark at 37°C for a minimum of 24 h until the individual positive colony appeared. The positively stained colony was picked and seeded into 10 ml of LB medium (Solarbio, L1010) for 37°C, 200 rpm incubation. When the CFU (Colony Forming Unit) of the LB medium reached 1.8 × 10^10^ CFU/ml, the bacteria were harvested by centrifuging under 9000 *g* for 10 min. The bacterial pellets were washed twice in PBS and diluted to the concentration corresponding to 2 × 10^9^ CFU/kg body weight of each recipient WT mouse. To ensure the enriched bacteria is Shiga toxin‐producing *E. coli* (STEC), about 100 ml bacterial suspension was boiled for 10 min and centrifuged at 13,800 *g* for 10 min to gain the genome DNA and qPCR assay was used to detect *Stx1*, *Stx2*, and *eaeA*.

STEC suspension (100 μl) was administrated by oral gavage to 3‐month‐old C57BL/6J male mice after 8 h of starvation for food. Control animals received 100 μl of sterile PBS. After 4 h of ingestion of the bacterial suspension, both food and water were provided to the mice ad libitum. At 96 h after infection, mice stools were collected to detect *E. coli*. After 1 month, the same STEC transplantation assay was operated. In another month, feces and organs were harvested for further analysis.

### 2.6 Fecal water content assay

Three to five fecal pellets of each mouse were randomly selected, and the fecal water content of each fecal pellet was measured. The average fecal water content of each mouse was calculated and then presented in histograms. Fecal water content was measured as previously described (Jeong et al., 2017; Wang et al., 2020). Briefly, fecal pellets were collected into pre‐weighed petri dishes without lids, and the weight of each pellet was measured as the wet weight. Then, the pellets were dried at 80°C for 24 h, and the dry weight was measured. The water content of each fecal pellet was calculated according to the following equation:

fecal water content=1−fecaldryweightfecalwetweight×100%

### 2.7 qPCR

Total RNA was isolated from tissues after processing with TRIzol reagent (Invitrogen Life Technologies) following the manufacturer's instructions. RNA concentrations and purity were estimated by determining the A260/A230 and A260/A280 ratio with a Thermo Scientific Nanodrop 2000c (Thermo). Reverse transcription of mRNA was performed using the cDNA Synthesis Kit (TIANGEN). The kit contains gDNase which can efficiently remove genomic DNA, thus avoiding the interference of genomic DNA in Total RNA. PCR was carried out using SYBR Green (Yeasen) with CFX Manager 3.1 (Bio‐Rad). Each sample was processed in triplicate and normalized to GAPDH or β‐actin levels by the 2^−ΔΔCT^ method, and the values were expressed relative to those of the control group. Primers were ordered from Invitrogen, and sequences were shown in Table S3.

For bacterial quantification, DNA from mouse feces was extracted using QIAamp Fast DNA Stool Mini Kit (QIAGEN) according to the instructions. A 20 ng DNA sample was used for qPCR reactions using specific primers (Table S3) to amplify bacterial 16S rRNA. Bacterial abundance was normalized with the abundance of fecal total bacteria using the conserved eubacterial 16S rRNA primer pair. Results are presented as relative quantification.

For bacterial translocation analysis, spleen, kidney, and liver tissues were weighed and genomic DNA was purified using TIANamp Genomic DNA Kit according to the manufacturer's protocol (TIANGEN). *E. coli* gene levels were determined by qPCR and normalized by the conserved eubacterial. Data were analyzed by relative quantification.

### 2.8 Enzyme‐linked immunosorbent assay

Mouse serum and fecal samples were assessed by ELISA according to the kit manufacturers' instructions as follows: LPS, LCN2, IL‐1β, TNFα, and C‐reactive protein (CRP; LCN2: Cloud Clone Corp.; Others: CUSABIO). For the fecal sample, 40 mg of sample was first added to 0.2 ml PBS and pipetted thoroughly for 1 min; it was then balanced at room temperature (20–25°C) for 10 min and finally centrifuged at room temperature at 6000–8000 RCF for 10 min. The supernatant was collected into a clean 1.5 ml tube for ELISA measurements. Serum samples were assessed based on the concentration and the instructions from the kit. Measurements were generally performed by adding 50 μl of supernatant and 50 μl detection antibody to each well, and samples were then tested according to the procedures stated in the manual from the kit. Raw data from standard curves and sample wells were optimized and analyzed using a GLOMAX Multi Detection System (Promega).

### 2.9 16S rRNA sequencing analysis

Microbial DNA was extracted from fecal samples, and the 16S rRNA gene V4 region (515F‐806R) of the isolated DNA with the barcode was amplified by PCR and sequenced using the Illumina HiSeq platform (service provided by Novogene Corporation) following the manufacturer's guidelines. Primer sequences were 515F: 5′‐GTGCCAGCMGCCGCGGTAA, 806R: 5′‐GGACTACHVHHHTWTCTAAT. All PCR reactions were carried out in 30 μl reactions with 15 μl of Phusion® High‐Fidelity PCR Master Mix (New England Biolabs); 0.2 μM of forward and reverse primers, and about 10 ng template DNA. Thermal cycling consisted of initial denaturation at 98°C for 1 min, followed by 30 cycles of denaturation at 98°C for 10 s, annealing at 50°C for 30 s, and elongation at 72°C for 30 s and finally at 72°C for 5 min. USEARCH v10.0.240 was used for quality assurance and OTU picking for the raw sequences (Edgar, 2013). In brief, the raw sequences were first demultiplexed. Then, demultiplexed reads were merged into paired reads, and the primer was stripped based on Vsearch (2.14.2). Merged reads with expected error thresholds larger than 1.0 or read lengths shorter than 160 were discarded as quality filtration. The Amplicon Sequence Variants non‐clustering denoising was performed by Unoise3 of Usearch10. The quality‐filtered reads were dereplicated into unique sequences. Based on the abundance of the unique sequences, singletons were discarded. Then, the sequences were subjected to OTU clustering at 97% similarity. A chimera filter was built in this OTU clustering implementation based on the Silva database. After mapping all merged sequences picked by UPARSE to OTUs, a table was constructed. The OTU table was then subjected to QIIME 1.9.1 analysis (Kuczynski et al., 2011). The phylogenetic information of the OTUs was obtained using RDP classifier 11.5 with reference sequencing from 16S rRNA training set 16 of the Ribosomal Database Project using a bootstrap cutoff of 0.6 (Wang et al., 2007).

The gut microbiota diversity analysis and species taxonomy were based on alpha diversity, and beta diversity, shown as UniFrac distance, displayed in the PCoA plot and LEfSe.

### 2.10 Short‐chain fatty acid analysis

Short‐chain fatty acid analysis was performed as described (Zhao et al., 2006). Fecal pellets from each mouse sample were weighted, and approximately 100 mg was homogenized in 1 ml deionized water with 50% aqueous acetonitrile for 3 min using a stainless‐steel bar. Next, the SCFAs were extracted by vortexing for 5 min. Then, the pH of the suspension was adjusted to 2–3 and the suspension was subsequently transferred to a polypropylene tube and centrifuged at 3000 *g* for 20 min at 10°C, after that the clear supernatant was collected. Chemical derivatization was performed by mixing 20 μl of 200 mM 3NPH in 50% aqueous acetonitrile and 20 ml of 120 mM EDC‐6% pyridine solution with 40 μl of the supernatant after sample preparation. The mixture was reacted at 40°C for 30 min and was then dried with a Speedvac™ Vacuum Concentrator (Thermo) and stored at −80°C in a freezer. The fecal supernatant was spiked with a standard solution in advance. Finally, distillates of the sample material were analyzed with the Exactive™ GC Orbitrap™ GC–MS system (Thermo) supported by the Metabolomics Core Facility Platform, Tsinghua University, and SCFAs including acetate, formate, propionate, isobutyrate, butyrate, isovalerate, and valerate, with >50% above the limit of detection were assessed.

### 2.11 Western blot

To obtain proteins, the tissue mash was lysed in RIPA buffer (Biomiga) containing a protease inhibitor cocktail (AbMole Bioscience) and phosphatase inhibitors (Solarbio). The protein concentrations were measured using a BCA protein assay kit (Solarbio), following the instructions provided by the manufacturer. Then, proteins (50 μg/sample) were mixed with 6× loading buffer (Solarbio) and boiled for 5 min to denature them. Next, a 12% sodium dodecyl sulfate‐polyacrylamide gel was prepared in accordance with standard protocols. Standard electrophoresis was then performed, and proteins were transferred onto polyvinylidene difluoride membranes (Millipore). The membrane was blocked with 5% skim milk and 0.1% fetal calf serum at 37°C for 1 h. Then, it was incubated with primary antibodies against Sirt6 (1:1000, ab62739, Abcam), p16 (1:1000, ab51243, Abcam), p21 (1:1000, ab188224, Abcam), β‐actin (1:1000, 4970S, Cell Signaling Technology), and GAPDH (1:1000, ab181602, Abcam) overnight. After incubation, the blot was washed three times with TBST and then incubated with TBST containing a 1:1000 dilution of horseradish peroxide‐conjugated goat anti‐rabbit antibody (Abcam) for 2 h at room temperature. After washing with TBST three times, the blot was developed with an ECL kit (Sigma) and visualized by Chemi Capture (CLINX). Images were taken, and gray statistics were analyzed using Imaging Lab software.

### 2.12 Histological staining

All tissues were fixed in 4% paraformaldehyde (Servicebio) for 24 h at 4°C and then embedded in paraffin. The samples were dehydrated, and 4 μm sections were studied. H&E staining, PAS staining, and Alcian blue staining were performed according to the manufacturer's instructions (Servicebio). Photographs were captured using a light microscope (Axio Scan.Z1). Zen 2.3 (blue edition, Carl Zeiss Microscopy GmbH, 2011) was used for the morphological assessment based on a scoring system in a blinded manner as previously reported (Xu et al., 2020). Intestinal villi lengths were measured by using ImageJ (Fiji). Use the “Analyze‐Set Scale” to set the measuring scale, then use the straight‐line tool to measure the intestinal villi lengths. Three fields of each section were randomly selected, and then, all villi in this field were measured and the average villi length of each field was calculated. The average length of each field was presented in histograms.

The severity of inflammation (with a score ranging from 0 to 3, indicating no inflammation, mild, moderate, or severe), mucosal damage (with a score ranging from 0 to 3, indicating none, mucosa, submucosa, transmembrane), and crypt damage (with a score of 0 to 4, indicating none, one‐third of basal are damaged, basal two‐third are damaged, only epithelium is intact, and the entire crypt and epithelium are lost) were independently measured to access the histological score of colon and small intestine. Each parameter scored was multiplied by the percentage of tissue involved, and the total was added up to obtain the histopathological score. Three fields of each section were randomly selected and scored. The average histological score of each section was calculated and then presented in histograms.

### 2.13 Immunohistochemistry staining

Immunohistochemistry (IHC) was performed on formalin‐fixed, paraffin‐embedded tissue. 5 μm paraffin sections were cut. After antigen retrieval with citrate solution, slides were rinsed and blocked with a peroxidase‐blocking reagent and incubated with p16^INK4a^ antibody or p21^WAF1^ antibody (ab51243 and ab188224, Abcam). Immunoreactive signals were visualized with DAB Quanto chromogen (Servicebio). Then, slides were counterstained with hematoxylin, dehydrated, mounted, and covered with a coverslip. Immunoreactivity was visualized by a light microscope (Axio Scan.Z1). Images were analyzed using ImageJ (NIH, version 2.2.0/1.53c). p16 and p21 were quantified using a color deconvolution algorithm to identify DAB positivity in defined ImageJ‐based macros regions of interest (ROI) for each field. Specific ROI was selected to exclude inappropriate regions. Those regions without nucleus‐positive staining co‐located were regarded as false‐positive regions. When calculating, these regions were excluded specifically. Percentages of positive cells were calculated for each field. Three fields of each section were randomly selected and measured. The average percentage of each section was calculated and then presented in histograms.

### 2.14 SA‐β‐gal activity assay

SA‐β‐gal staining was performed in accordance with the manufacturer's instructions (Servicebio, G1073). Briefly, frozen tissues (8 μm thick) were rewarmed at room temperature for 10 min and then fixed in fix‐solution for 20 min at room temperature. The frozen sections were washed three times with PBS and then incubated with SA‐β‐gal staining solution (pH 6.0) overnight at 37°C without CO_2_. After completion of SA‐β‐gal staining, the sections were counterstained with eosin for 5 min and then rinsed with ddH_2_O three times. Sections were dehydrated in absolute alcohol two times and cleared in xylene for 5 min. Excess xylene was removed, and a coverslip was placed over the section. After drying overnight at 4°C, the sample was observed and visualized by a light microscope (Axio Scan.Z1). Three fields of each section were randomly selected and measured. The average percentage of each section was calculated and then presented in histograms. Liver, spleen, and kidney frozen sections stained with SA‐β‐gal were quantified by ImageJ software (NIH, version 2.1.0/1.53c) to measure the positive staining area. The total area was quantified by the eosin‐positive area. The relative SA‐β‐gal‐positive area was calculated with the SA‐β‐gal‐positive area divided by the total area. “Color threshold” was used to select the target area, for the SA‐β‐gal‐positive area, the threshold was set as Red (0,100), Green (0,20), and Blue (0,200); for the eosin‐positive area, the threshold was set as Red (100,255), Green (0,58), and Blue (140,255). “Measure” restricted to threshold was used to measure the area. For the statistics of the SA‐β‐gal‐positive area of the liver and spleen, the regions were randomly selected to be photographed. The SA‐β‐gal‐positive area of the kidney was randomly selected in the regions avoiding renal proximal tubular epithelium to avert false positives.

### 2.15 Detection and discrimination of *E. coli* strain

Fecal samples from SIRT6 KO mice (20 mg) were homogenized in 1 ml of Trypto‐casein‐soy (TCS) broth (Solarbio). The fecal stock was diluted 100 times, 1000 times, and 10,000 times, respectively, using TCS broth and incubated at 37°C for 3 h. 100 μl aliquots were plated on *E. coli* chromogenic media (Solarbio) and incubated at 37°C for 20 h in aerobic conditions. *E. coli* chromogenic media is a selective and differential medium for the isolation of all *E. coli* and several non‐fermenting Gram‐negative bacteria. The overnight cultures were examined for bacterial growth and colony morphology. Aquamarine blue colonies on the chromogenic medium were *E. coli*. DNA was extracted from randomly selected aquamarine blue colonies and directly tested for the *aggR*, *eaeA*, *Stx1*, *Stx2*, *St (1a/1b)*, *It, ipaH*, and *daaD* genes by qPCR. Primer sequences were listed in Table S3.

### 2.16 FITC‐dextran intestinal permeability assay

Gut permeability was measured using Fluorescein isothiocyanate (FITC)‐dextran (average molecular weight: 4000 Da). Briefly, mice fasted for 6 h were intragastric administration with FITC‐dextran (600 mg/kg body weight, 180 mg/ml). After 4 h, blood samples were collected and centrifuged (3000 *g* at 4°C) for 10 min, and serum was collected. GLOMAX Multi Detection System (Promega) was used to determine FITC‐dextran concentration. Standard curves were produced by serial dilution of FITC‐dextran in serum from untreated mice.

### 2.17 Statistical analysis

Results are expressed as means ± SEMs. Statistical significance was evaluated using a two‐tailed unpaired *t*‐test, one‐way ANOVA, or two‐way ANOVA with Tukey's multiple comparisons test. PERMENOVA tests were used for PCoA analysis. A *p*‐value less than 0.05 was considered significant. Data were analyzed and plotted in Graph Pad Prism 9.0 software, STAMP software, or R version 3.6.2.

## 3 RESULTS

### 3.1 SIRT6 KO mice house gut dysbiosis, and transferring the microbiota from KO to WT mice confers premature aging phenotypes

**FIGURE 1:**
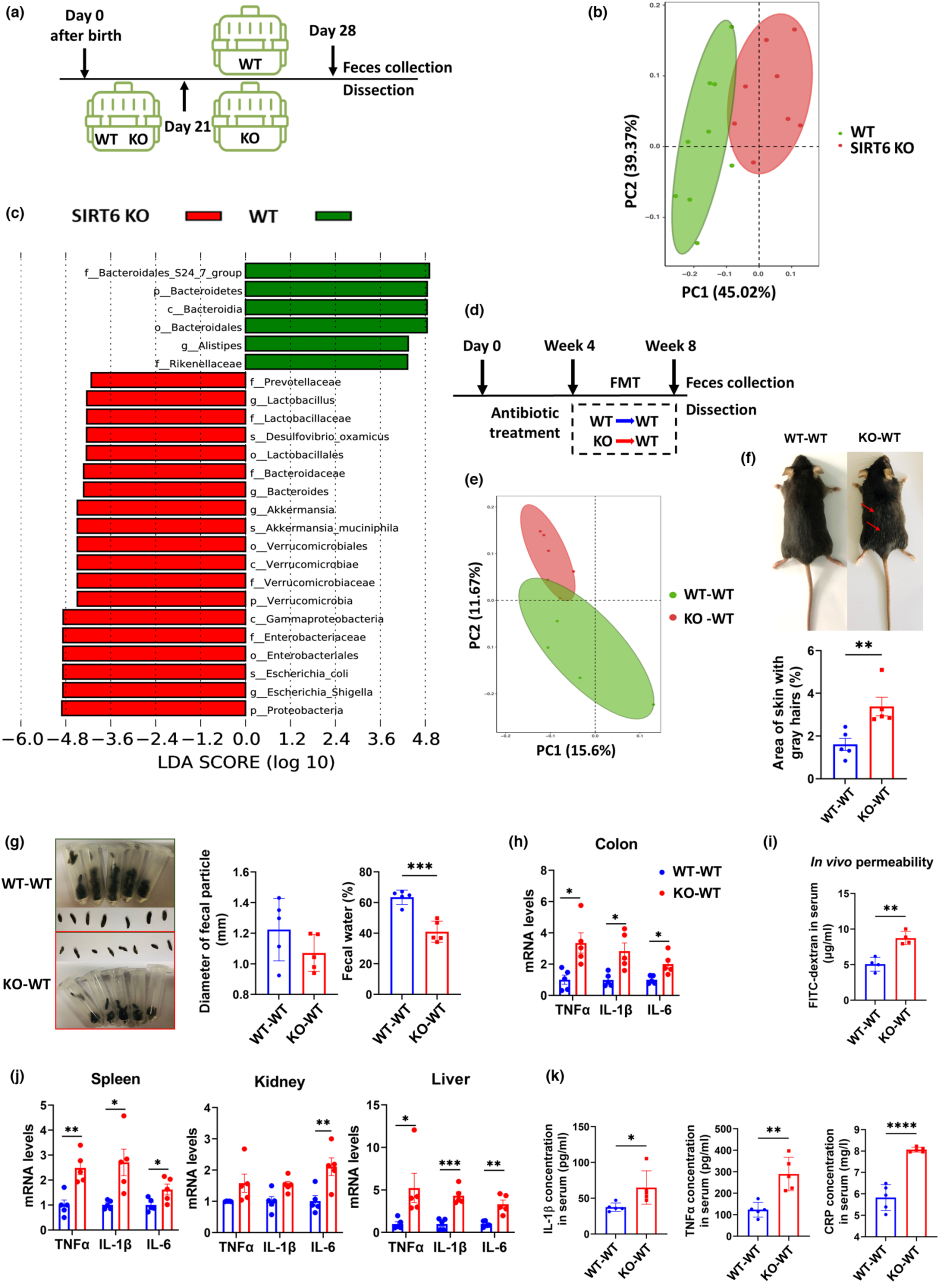
Gut dysbiosis of SIRT6 KO mice drives premature aging phenotypes in recipient WT mice through FMT. (a) Scheme of experimental design, in which the difference of gut microbiota between WT and SIRT6 KO mice (129S6/SvEvTac, 129Sv) was analyzed: Mice were individually caged from Day 21 after birth and were harvested at Day 28. (b) Principal Coordinates Analysis (PCoA) plot of weighted UniFrac distance (*p* = 0.001; AMOVA) between WT and SIRT6 KO mice. Each dot represents an individual mouse (WT, *n* = 9; SIRT6 KO, *n* = 8). (c) Results of LEfSe analysis show bacterial taxa compositions that were significantly different between SIRT6 KO and WT mice. LDA, linear discriminant analysis (WT, *n* = 9; SIRT6 KO, *n* = 8). (d) Experimental design of FMT. WT‐WT (C57BL/6J WT mice transplanted with fecal microbiota from 129Sv WT mice) and KO‐WT (C57BL/6J WT mice transplanted with fecal microbiota from 129Sv SIRT6 KO mice). Mice were treated for 4 consecutive weeks with an antibiotic cocktail in drinking water and then were given the microbiota suspension every other day for 4 weeks. Feces for further analysis were collected 72 h after FMT. (e) PCoA of weighted UniFrac distances between WT‐WT and KO‐WT mice (*p* = 0.243; PERMANOVA). Each dot represents an individual mouse (*n* = 5 per group). (f) Representative pictures of WT‐WT mice and KO‐WT mice. KO‐WT mice showed more gray hairs. More replicates were shown in Figure S3a. The area of skin gray hairs was quantified using Image J (*n* = 5 per group). (g) Appearances of feces. The diameter and water content of feces from WT‐WT and KO‐WT mice were measured (*n* = 5 per group). Each dot represents an individual mouse. (h) Relative mRNA expression of inflammatory factors, including TNFα, IL‐1β, and IL‐6, measured by qPCR, in colon tissue (*n* = 5 per group). (i) FITC‐dextran in vivo permeability assay in WT‐WT and KO‐WT mice (*n* = 4 per group). (j) Relative mRNA expression of inflammatory factors, including TNFα, IL‐1β, and IL‐6, measured by qPCR in the spleen, kidney, and liver (*n* = 5 per group). (k) Serum IL‐1β, TNFα, and CRP concentration (*n* = 5 per group). *p < 0.05, **p < 0.01, ***p < 0.001, ****p < 0.0001

To characterize the presence and level of gut dysbiosis in SIRT6 KO mice, we collected fecal samples from SIRT6 KO mice and WT littermates (Figure 1a). Western blot analysis of the total colon tissue protein confirmed that SIRT6 was absent in KO mice (Figure S1a). We observed increased intestinal pathological damage (Figure S1b) and inflammation (Figures S1c,d), indicating that spontaneous colitis occurred in SIRT6 KO mice (Mostoslavsky et al., 2006). We then performed 16S rRNA gene profiling and found two distinct groups of microbiomes by principal coordinate analysis (PCoA; weighted, Figure 1b; unweighted, Figure S1e). A significant decrease in operational taxonomic units (OTUs) was observed, from 1419 in WT mice to 1190 in SIRT6 KO mice (Figure S1f). Indeed, we observed a sharp reduction in the concentrations of SCFAs, particularly acetate and butyrate, in SIRT6 KO mice, indicating gut dysbiosis (Figure S1g). This result was supported by the lower overall abundance of Bacteroidetes (Figure S1h), widely recognized for their ability to digest complex dietary compounds and improve the yield of energy harvested from food (Khan Mirzaei et al., 2020). Accordingly, we also identified a slight but insignificant decrease in the abundance of key SCFAs‐producing taxa, including Ruminococcaceae and Lachnospiraceae (Figure S1i). SIRT6 KO mice were substantially enriched in pro‐inflammatory bacterial taxa, such as Enterobacteriaceae (Figures 1c and S1j; Fachi et al., 2019; Sassone‐Corsi et al., 2016). Moreover, Verrucomicrobiaceae, Proteobacteria, and Prevotellaceae, known to be enriched in progeria mice and patients (Bárcena et al., 2019), were also significantly abundant in SIRT6 KO mice (Figures 1c and S1j). We also found a marked increase in the bacteria of the *Helicobacter* genus (including *Helicobacter typhlonius*, *Helicobacter bilis*, and *Helicobacter hepaticus*), *Enterococcus*, and *Bacteroides*, and a reduction in *Faecalibacterium prausnitzii* (Figure S1j; Hale et al., 2007; Ni et al., 2017; Shin et al., 2015). Together, these data indicate that SIRT6 KO mice can be characterized by the presence of severe gut dysbiosis.

We performed FMT to explore whether gut dysbiosis contributes to the aging phenotype. We used SIRT6 KO mice or WT mice as microbiome donors and young WT mice as recipients, abbreviated as KO‐WT and WT‐WT, respectively (Figure 1d). In addition, we included a group of WT mice without any intervention as controls. FMT effectiveness was evaluated by comparing the gut microbiota profiles of donors and recipients; this analysis showed that the transplanted WT mice acquired donor microbiota (Figures S2a,b). We found a difference in the beta diversity index indicated by the PCoA plot, decreased OTUs, and decreased alpha diversity indicated by the Chao1 and ACE indices in KO‐WT mice (Figures 1e and S2c,d). KO‐WT mice showed coarser and more gray hair after 4 weeks of FMT (Figures 1f and S3a). While KO‐WT mice maintained normal body weight, they had lower serum glucose levels (Figures S3b,c). Notably, hypoglycemia is widely considered to be one of the main causes of death in SIRT6 KO mice (Mostoslavsky et al., 2006; Xiao et al., 2010). We observed mild fat loss in KO‐WT mice (Figure S3d; Table S1), accompanied by smaller lipid droplets in white and brown adipose tissue (Figure S3e), consistent with adipose tissue atrophy in SIRT6 KO mice (Li et al., 2020). The above data show that gut microbiota from KO mice can affect lipid metabolism in their recipients and that KO‐WT mice partly mimic pathological features of SIRT6 KO donor mice, suggesting that SIRT6 deficiency phenotypes can be transferred by gut microbiota. Considering that the intestines are the primary organs affected by the gut microbiota, we next sought to understand how FMT influences intestinal barrier function. Interestingly, we observed shriveled fecal particles, with decreased diameter and water content, in KO‐WT mice (Figure 1g), similar to those found in SIRT6 KO mice. qPCR analysis of colonic tissue revealed elevated levels of inflammatory factors, including TNFα, IL‐1β, and IL‐6, in KO‐WT mice (Figure 1h). We also observed damaged colon and small intestine architecture in KO‐WT mice, indicated by the evaluation of the histological score, villus length, and mucus glycoproteins (Figure S4a). Genes involved in the formation of epithelial junctions in the intestine, including claudin‐1, occludin, and ZO‐1, were downregulated (Figure S4b). Serum endotoxins, generally referred to as lipopolysaccharides (LPS), are commonly used as indirect indicators of intestinal permeability (Vancamelbeke & Vermeire, 2017). Serum LPS concentrations were significantly higher in KO‐WT mice than in WT‐WT mice (Figure S4c). We also performed a gut permeability assay and found that intestinal permeability was increased in KO‐WT mice (Figure 1i). Together, our findings suggest that fecal microbiota transplanted from SIRT6 KO mice can promote intestinal inflammation and disrupt intestinal barrier function in recipient mice.

**FIGURE 2:**
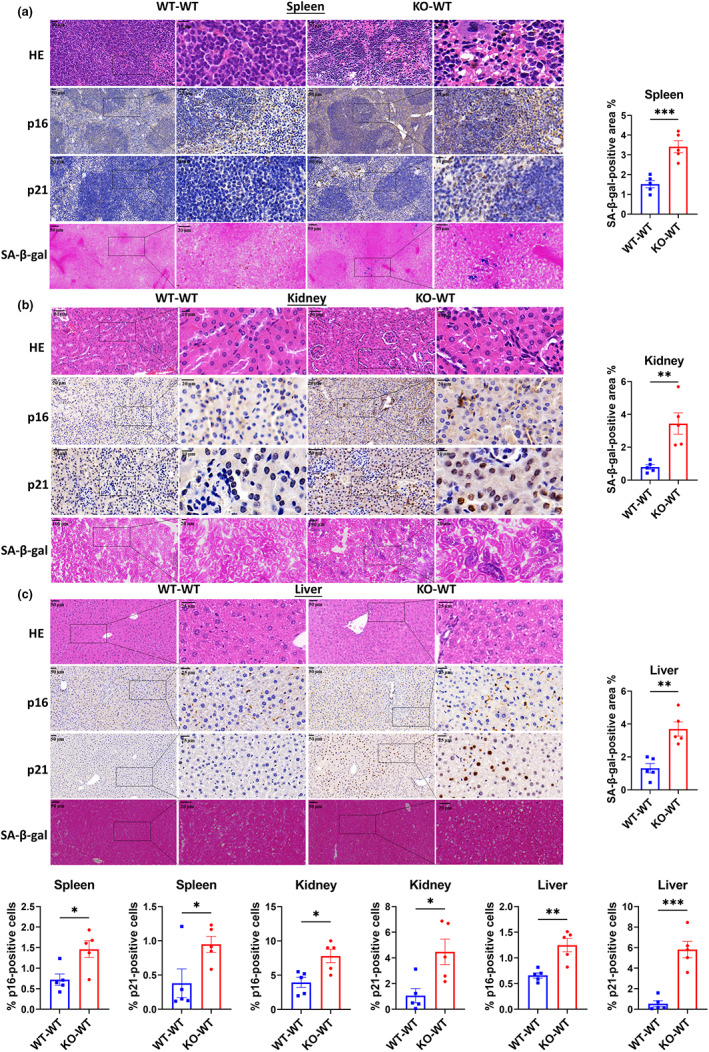
FMT of SIRT6 KO donor microbiomes into WT recipients results in visceral senescence. Representative sections stained with H&E (HE), immunohistochemistry of p16 and p21 antibody, and SA‐β‐gal in the spleen (a), kidney (b), and liver (c) tissues (*n* = 5 per group). Each dot represents an individual mouse. *p < 0.05, **p < 0.01, ***p < 0.001

We also observed aging‐associated changes in the spleen, kidney, and liver, commonly used to measure biological aging (Lee et al., 2013; Levitsky, 1980). There was a significant increase in the expression of inflammatory markers in the spleen, kidney, and liver of KO‐WT mice (Figure 1j). Additionally, high levels of serum IL‐1β, TNF‐α, and CRP, as well as enlarged spleens, indicated that the presence of inflammation in KO‐WT mice was systemic (Figures 1k and S3d). Systemic inflammation has been reported to enhance fat loss and hypoglycemia (Fischer et al., 2015; Gruther et al., 2008; Kealy et al., 2020), suggesting that the observed reductions in fat mass and serum glucose levels may be due to the increased inflammatory state in KO‐WT mice. P16^lnk4a^ (p16) and p21^waf1^ (p21) accumulate in various tissues during normal aging in rodents and humans and are thus used as senescence markers (Krishnamurthy et al., 2004; Yousefzadeh et al., 2020). We observed that the mRNA levels of these markers were increased in the colon (Figure S4d), whereas protein levels were increased in the spleen, kidney, and liver, as observed by immunohistochemistry and Western blot (Figures 2a–c, and S4e). However, increased expression of these markers can also be caused by active macrophages. Therefore, we performed a β‐galactosidase staining assay and found an increased β‐galactosidase positive area ratio in different tissues of KO‐WT mice compared to WT‐WT control mice, directly indicating visceral senescence in KO‐WT mice (Figures 2a–c). Here, we show that gut microbiota from SIRT6 KO mice induces inflammation and cell senescence when transplanted into WT mice, suggesting that gut dysbiosis is likely to contribute to premature aging phenotypes in SIRT6 KO mice.

**FIGURE 3:**
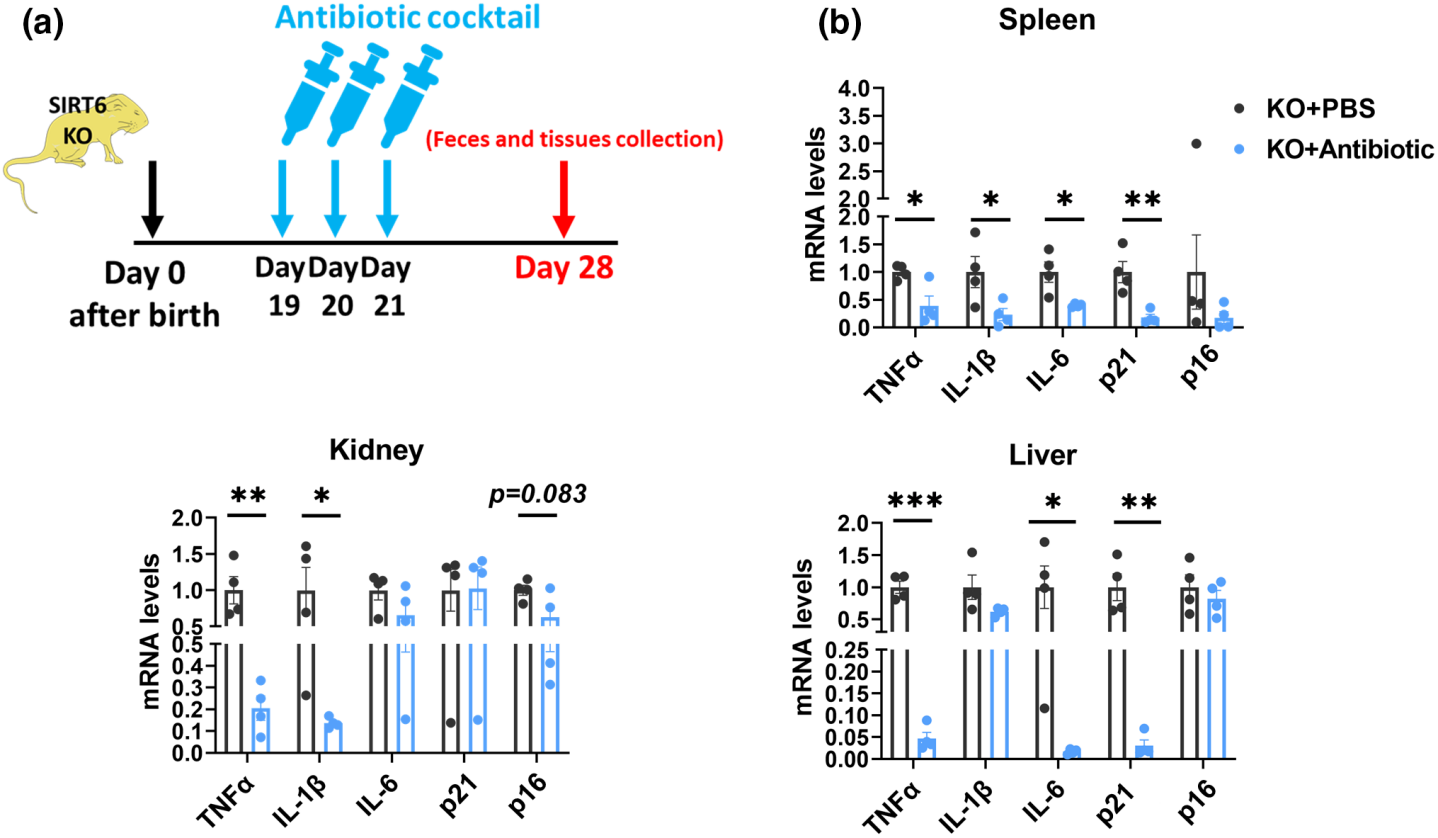
Antibiotic treatment attenuates cell senescence of SIRT6 KO mice. (a) Experimental design of antibiotic treatment. Nineteen‐day‐old KO mice (129Sv) were administered 100 μl of an antibiotic cocktail containing 1 g/L ampicillin, 1 g/L neomycin, 1 g/L metronidazole, and 0.5 g/L vancomycin hydrochloride for 3 days. Feces and tissues were collected when mice were 28 days old. (b) Relative mRNA expression of inflammatory factors including TNFα, IL‐1β, and IL‐6, and senescence markers including p21 and p16 in the spleen, kidney, and liver tissues from KO + PBS and KO+ Antibiotic mice were measured by qPCR (*n* = 4 per group). *p < 0.05, **p < 0.01, ***p < 0.001

To confirm the causative link between gut dysbiosis and premature aging phenotypes in SIRT6 KO mice, we administered an oral antibiotic cocktail to 19‐day‐old KO mice for 3 consecutive days (Figure 3a), and collected feces and tissues when mice were 28 days old. Consistently, we also found that KO + Antibiotic mice had decreased expression of inflammatory factors including TNFα, IL‐1β, and IL‐6, and senescence markers including p16 and p21 (Figure 3b). These data provide direct evidence of causality to claim that gut dysbiosis contributes to the premature aging phenotype in SIRT6 KO mice.

### 3.2 Gut microbiota from WT mice extends lifespan and improves gut dysbiosis in SIRT6 KO mice

**FIGURE 4:**
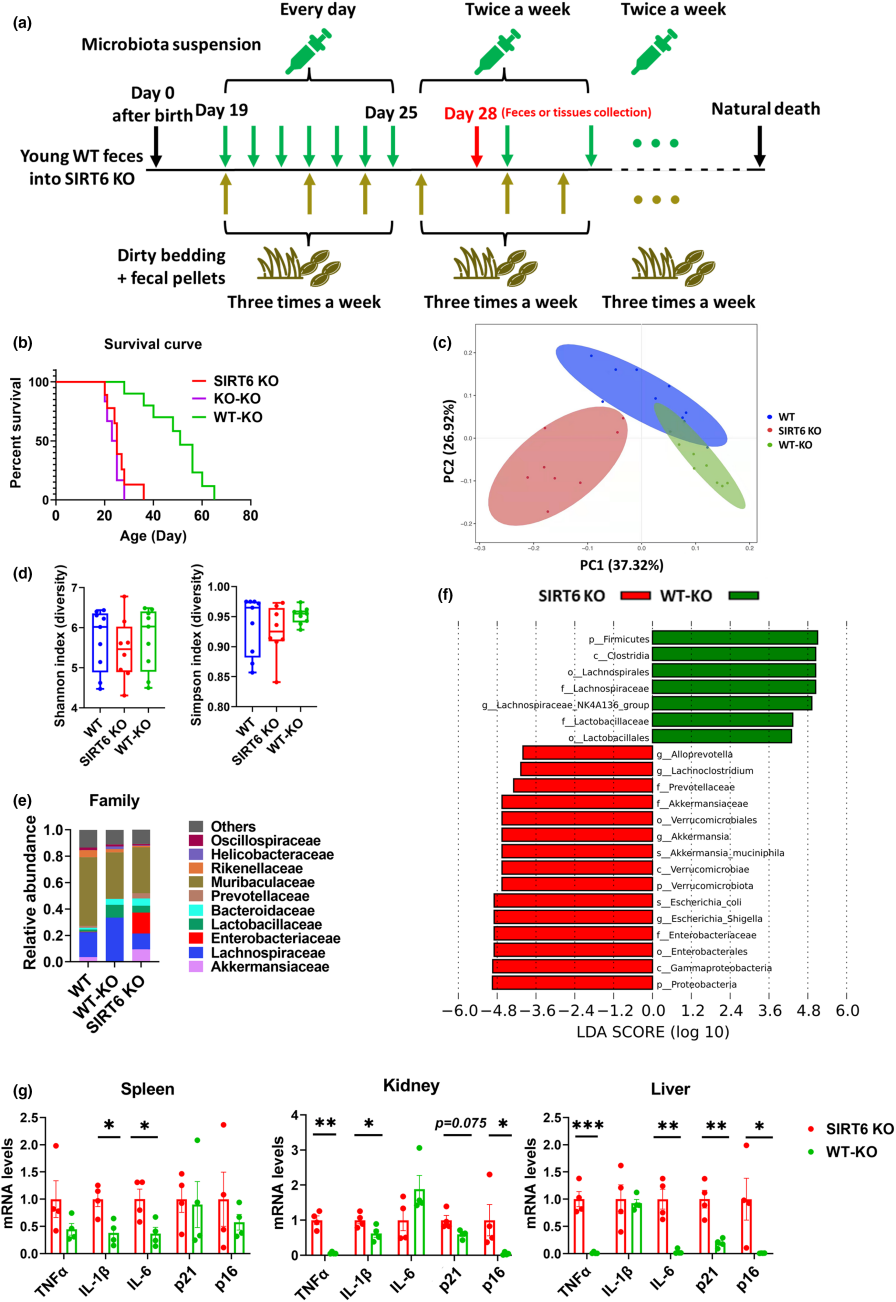
FMT of WT donor microbiomes into SIRT6 KO recipients results in extended lifespan and improved gut dysbiosis. (a) Experimental design of FMT. FMT started when SIRT6 KO mice were 19 days old without antibiotic treatment. Microbiota suspension was carefully administered orally to mice. SIRT6 KO mice were given microbiota suspension per day for 1 week. After 1 week, mice received the microbiota suspension twice a week until natural death. During FMT, cages of recipient mice (SIRT6 KO) were replenished with dirty bedding and fecal pellets from WT mice three times a week. Feces or tissues were collected when mice were 28 days old, or lifespan was recorded. (b) Percentage survival of SIRT6 KO (129Sv), WT‐KO (129Sv SIRT6 KO mice transplanted with fecal microbiota from 129Sv WT mice), and KO‐KO mice (129Sv SIRT6 KO mice transplanted with fecal microbiota from 129Sv SIRT6 KO mice; SIRT6 KO, *n* = 8; WT‐KO, *n* = 9; KO‐KO, *n* = 6). (c) Principal coordinate analysis of weighted UniFrac distances (*p* < 0.001; PERMANOVA). Each dot represents an individual mouse (SIRT6 KO, *n* = 8; WT‐KO, *n* = 9; WT, *n* = 9). (d) Alpha diversity was analyzed based on the Shannon and Simpson indices (SIRT6 KO, *n* = 8; WT‐KO, *n* = 9; WT, *n* = 9). (e) Average relative abundance of the top 10 most abundant bacteria at the family level in WT, SIRT6 KO, and WT‐KO mice (SIRT6 KO, *n* = 8; WT‐KO, *n* = 9; WT, *n* = 9). (f) Results of LEfSe analysis show bacterial taxa compositions that were significantly different between SIRT6 KO and WT‐KO mice (SIRT6 KO, *n* = 8; WT‐KO, *n* = 9). LDA, linear discriminant analysis. (g) Relative mRNA expression of inflammatory factors including TNFα, IL‐1β, and IL‐6, and senescence markers including p21 and p16 in the spleen, kidney, and liver tissues from KO and WT‐KO mice were measured by qPCR (*n* = 4 per group). *p < 0.05, **p < 0.01, ***p < 0.001

To explore whether improving gut microbiota can rescue shortened lifespan and attenuate premature aging phenotypes of KO mice, we performed FMT in SIRT6 KO mice using microbiota from WT mice (WT‐KO; Figure 4a). WT‐KO mice exhibited enhanced survival compared to KO‐KO mice (SIRT6 KO mice transplanted with microbiota from SIRT6 KO mice) and SIRT6 KO mice (Figure 4b). KO‐KO mice had a slightly worse survival rate than SIRT6 KO mice. Therefore, we continued to test the improvement in WT‐KO mice compared with SIRT6 KO mice, but not KO‐KO mice, to explore the benefits of FMT in SIRT6 KO mice. Feces were collected at 28 days of age, and 16S rRNA gene profiling was performed. We found a distinct difference in the beta diversity index and also slightly increased alpha diversity indicated by Shannon and Simpson indices, in WT‐KO mice compared to SIRT6 KO mice (Figures 4c,d). The effectiveness of FMT was evaluated by comparing the gut microbiota profiles of donors and recipients. This analysis showed that WT‐KO mice partially acquired the microbiome of WT mice (Figure 4e). LEfSe analysis demonstrated a loss of multiple pathogenic bacteria species in WT‐KO mice that were enriched in SIRT6 KO mice, including Enterobacteriaceae, Verrucomicrobiaceae, Proteobacteria, and Prevotellaceae (Figures 4e,f), suggesting an improvement in gut dysbiosis in WT‐KO mice. In addition, we collected organs and tissues from 28‐day‐old WT‐KO mice, and the mRNA levels of inflammatory factors and senescence markers were measured. WT‐KO mice presented decreased inflammation and cell senescence in different tissues such as the spleen, kidney, and liver (Figure 4g). These results demonstrate that FMT from WT mice can improve gut dysbiosis, enhance the lifespan, and attenuate inflammation and cell senescence in SIRT6 KO mice, supporting the role of the dysregulated microbiome in premature aging in SIRT6 KO mice.

### 3.3 Translocation of Enterobacteriaceae into visceral organs, enhanced by overgrowth of *E. coli*, drives aging‐associated features

**FIGURE 5:**
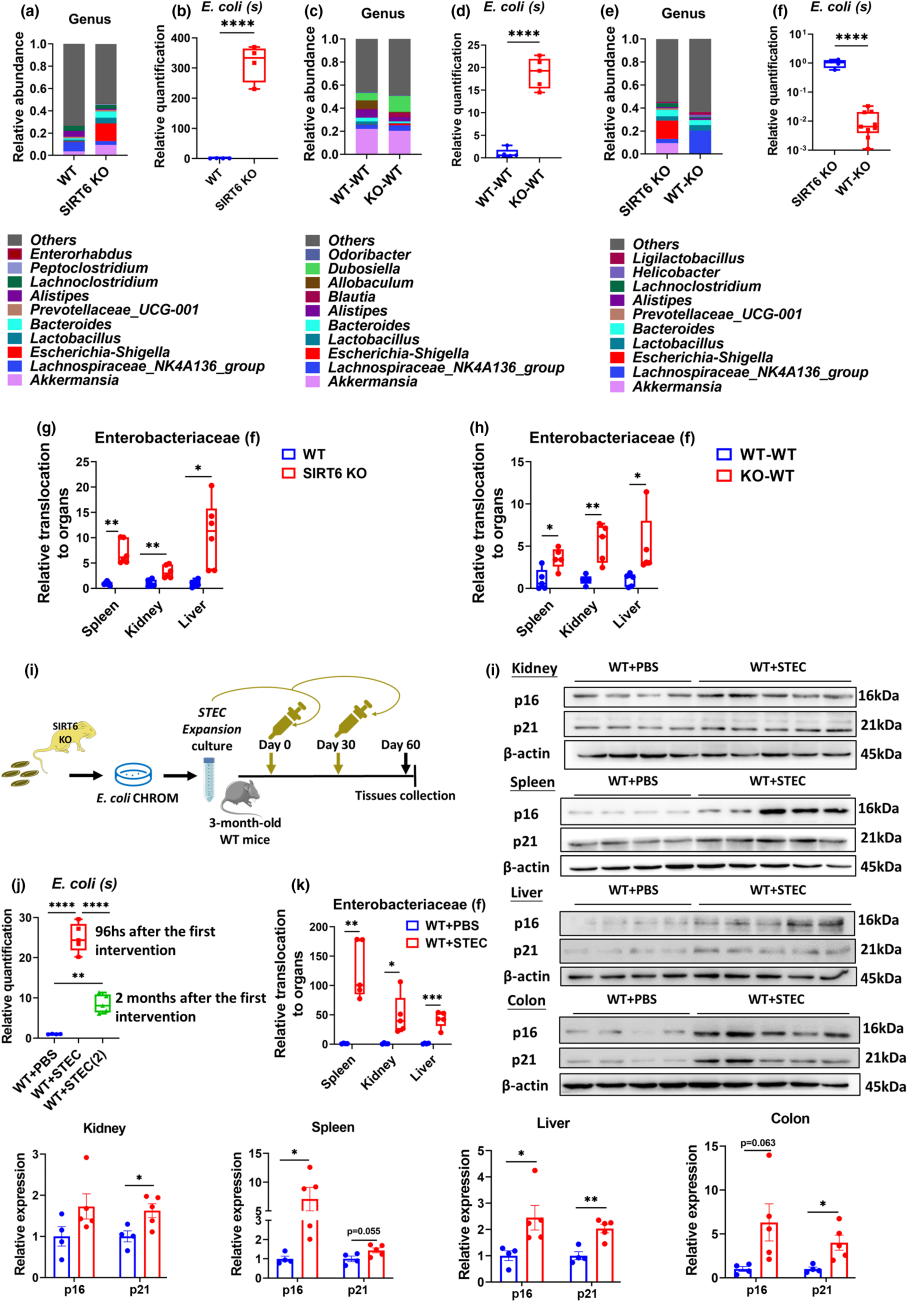
Validation of the aging‐promoting effects of *E. coli*. (a) Average relative abundance of the top 10 most abundant bacteria at the genus level in WT and SIRT6‐KO mice (WT, *n* = 9; SIRT6‐KO, *n* = 8). (b) Validation of the differences in the abundance of *E. coli* between WT and SIRT6‐KO mice by qPCR (*n* = 4 per group). (c) Relative abundance of the top 10 most abundant bacteria at the genus level in WT‐WT and KO‐WT mice (*n* = 5 per group). (d) Validation of the differences in the abundance of *E. coli* between WT‐WT and KO‐WT mice by qPCR (*n* = 5 per group). (e) Average relative abundance of the top 10 most abundant bacteria at the genus level in SIRT6 KO and WT‐KO mice (SIRT6 KO, *n* = 8; WT‐KO, *n* = 9). (f) Validation of the differences in the abundance of *E. coli* between SIRT6 KO and WT‐KO mice by qPCR (SIRT6 KO, *n* = 4; WT‐KO, *n* = 9). (g) Relative Enterobacteriaceae translocation of spleen, kidney, and liver between WT and SIRT6 KO mice was compared (*n* = 6 per group). (h) Relative Enterobacteriaceae translocation of spleen, kidney, and liver between WT‐WT and KO‐WT mice was compared (*n* = 5 per group). (i) Experimental design of mice administered *E. coli* by oral gavage. STEC, separated by *E. coli* chromogen medium (CHROM) from SIRT6 KO feces, were amplification cultured, and then orally supplemented, once a month and twice in all, to WT mice. Feces and tissues were collected for analysis. (j) Validation of the differences in the abundance of *E. coli* between WT + PBS and WT + STEC mice 2 months after transplantation by qPCR (WT + PBS, *n* = 4; WT + STEC, *n* = 5). (k) Relative Enterobacteriaceae translocation of spleen, kidney, and liver between WT + PBS and WT + STEC mice was compared (*n* = 5 per group). (l) Expression of p16 and p21 measured by Western blotting in kidney, spleen, liver, and colon tissues. Each dot represents an individual mouse in a statistical graph. (WT + PBS, *n* = 4; WT + STEC, *n* = 5). *p < 0.05, **p < 0.01, ***p < 0.001, ****p < 0.0001

The abundance of *Escherichia* (family Enterobacteriaceae) increases in an age‐dependent fashion and does not appear to be influenced by other factors such as medication or disease (Leite et al., 2021). When comparing the microbial population compositions of SIRT6 KO and WT mice, the most significantly enriched species was *E. coli*, which was over 300 times higher in SIRT6 KO mice than in WT mice, as validated by qPCR (Figures 5a,b). Increased *E. coli* abundance was also observed in recipient KO‐WT mice (Figures 5c,d). Moreover, the difference in the abundance of *E. coli* was sustained for 2 months after FMT (Figure S5a), indicating successful colonization after FMT. Notably, compared with SIRT6 KO mice, the bacteria with the greatest decrease in abundance was *E. coli* in WT‐KO mice (Figures 5e,f). The abundance of *E. coli* in the feces of KO mice could be reduced effectively with antibiotic treatment (Figure S5b). A high abundance of *E. coli* negatively affects the relative abundance of other probiotics as well as overall microbial diversity (Leite et al., 2021), suggesting that overgrowth of *E. coli* was likely the catalytic event exacerbating further dysbacteriosis and associated symptoms in SIRT6 KO mice.

The overgrowth of harmful gut microbes results in increased bacterial translocation and tissue damage (Nicolle et al., 2004). To investigate whether increased bacterial translocation during *E. coli* overgrowth contributes to premature aging, we measured the relative translocation of Enterobacteriaceae in the spleen, kidney, and liver tissues of KO mice using qPCR, and found an increase in Enterobacteriaceae load in KO mice (Figure 5g). Similarly, Enterobacteriaceae load in KO‐WT mice was also higher than that in WT‐WT mice (Figure 5h). These results indicate that dysbiosis may drive aging phenotypes in KO and KO‐WT mice through translocation of Enterobacteriaceae into visceral organs.

Positive PCR results for *Stx1*, *Stx2*, and *eaeA*, but negative results for other genes suggested that the Shiga‐toxigenic *E. coli* (STEC) strain was present in SIRT6 KO mice (Figure S5c; Guion et al., 2008). To further explore whether STEC contributes to the aging phenotypes, we cultured STEC extracted from the feces of SIRT6 KO mice, and orally administered these extracts suspended in PBS or PBS alone to 3‐month‐old WT mice (WT + STEC and WT + PBS, respectively; Figure 5i). Feces and different tissues were collected 2 months after the initiation of the intervention. We confirmed a higher abundance of *E. coli*, at 96 h and 2 months after the first intervention, in WT + STEC mice by qPCR (Figure 5j). Meanwhile, we measured the relative Enterobacteriaceae load in different tissues, and the results showed a greater Enterobacteriaceae translocation into the spleen, kidney, and liver tissues of WT + STEC mice (Figure 5k). Indeed, WT + STEC mice presented increased expression of p16 and p21 proteins, as well as β‐galactosidase positive area ratio, in a variety of tissues, including the kidney, spleen, liver, and colon (Figures 5l and S5d), suggesting the occurrence of cellular senescence in various organs in WT + STEC mice. Altogether, our data indicate that the overgrowth of *E. coli* enhances Enterobacteriaceae translocation which then accelerates certain aging phenotypes.

To test whether SIRT6 directly affected the gut microbiota, we collected and analyzed the feces from 4‐week‐old SIRT6 heterozygous (SIRT6+/−) mice and littermates WT mice. There was no difference in the abundance of feces Enterobacteriaceae and *E. coli* between SIRT6+/− and WT mice (Figure S6a). Moreover, SIRT6+/− mice harbored a healthy tissue structure and function supported by the normal histological score and PAS^+^ mucus area ratio of the colon and small intestine, as well as the villus length in the small intestine (Figure S6b). Inflammatory factors such as TNFα, IL‐1β, and IL‐6, and senescence markers such as p16 and p21 were not significantly different between SIRT6+/− and WT mice (Figure S6c). In summary, these data suggest that SIRT6+/− mice did not show gut dysbiosis, and therefore, that SIRT6 itself did not affect the gut microbiome.

### 3.4 Rebalancing of gut microbiota contributes to the high‐fat diet‐induced reversal of premature aging in SIRT6 KO mice

**FIGURE 6:**
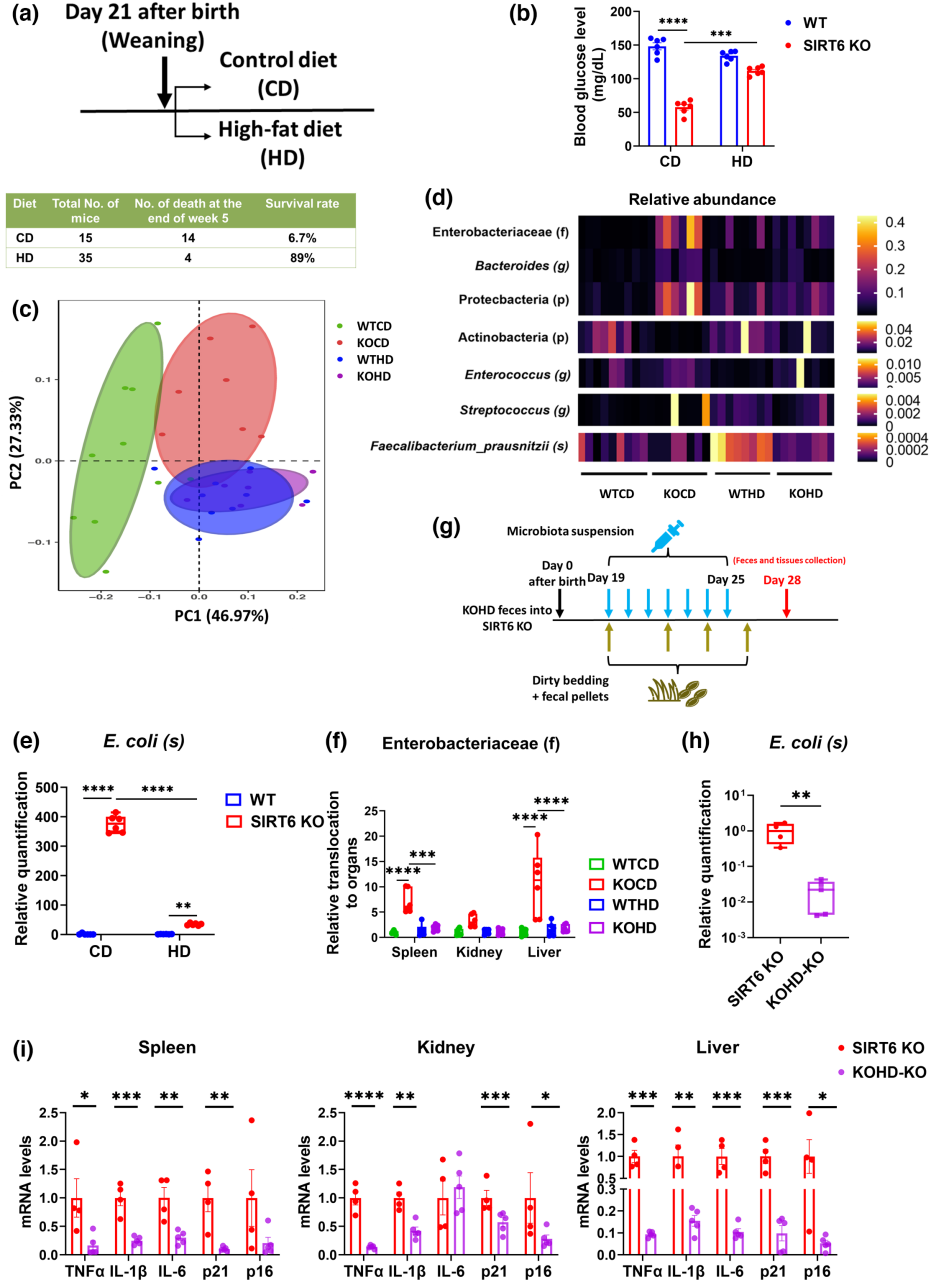
Gut dysbiosis of SIRT6‐KO mice is improved by a high‐fat diet. (a) Experimental design of high‐fat diet feeding. (129Sv WT mice fed with normal control diet), KOCD (129Sv SIRT6 KO mice fed with normal control diet), WTHD (129Sv WT mice fed with high‐fat diet), and KOHD (129Sv SIRT6 KO mice fed with high‐fat diet). SIRT6 KO mice were fed with CD or HD and caged individually from day 21 after birth. The survival rate was measured at the end of week 5. Notably, feces and tissue collection were performed at 4 weeks of age in WT and SIRT6 KO mice to ensure that enough SIRT6 KO mice were available for the experiment. (b) The blood glucose levels were tested after 3 h of fasting (*n* = 6 per group). (c) PCoA plot of weighted UniFrac distance (*p* < 0.001; PERMANOVA; Each dot represents an individual mouse (WTCD, *n* = 9; KOCD, *n* = 8; WTHD, *n* = 8; KOHD, *n* = 8). (d) Depiction of bacterial taxa whose abundance was associated with inflammation and aging (*n* = 6 per group). (e) Validation of the differences in the abundance of *E. coli* among four groups by qPCR (*n* = 6 per group). (f) qPCR analysis of relative *E. coli* load translocated to spleen, kidney, and liver (*n* = 6 per group). (g) Experimental design of FMT from KOHD mice. FMT started when SIRT6 KO mice were 19 days old without antibiotic treatment. Microbiota suspension was orally administered to mice. SIRT6 KO mice were given microbiota suspension once a day for 1 week. Cages of recipient mice (SIRT6 KO, 129Sv) were replenished with dirty bedding and fecal pellets from KOHD mice (129Sv) three times a week. Feces and tissues were collected at 28 days old. (h) Validation of the differences in the abundance of *E. coli* between KO and KOHD‐KO mice by qPCR (*n* = 5 per group). (i) Relative mRNA expression of inflammatory factors including TNFα, IL‐1β, and IL‐6, and senescence markers including p21 and p16 in the spleen, kidney, and liver tissues from KO (n = 4 per group) and KOHD‐KO mice (n = 5 per group) were measured by qPCR. *p < 0.05, **p < 0.01, ***p < 0.001, ****p < 0.0001

The above results highlight the role of gut dysbiosis in accelerating premature aging and suggest that gut microbiota can be targeted to improve aging phenotypes. Dietary intervention is one of the most effective strategies for restructuring the microbiome. We have previously reported that a high‐fat diet can reverse metabolic disorders and premature aging in SIRT6 KO mice (Li et al., 2020). This led us to wonder whether the benefits of a high‐fat diet result from gut microbiota alterations in SIRT6 KO mice. To address this question, we began feeding SIRT6 KO mice a high‐fat diet immediately after weaning (KOHD). Remarkably, 31 out of 35 (89% survival rate) lived past the end of the fifth week, compared with a 6.7% survival rate in the control diet group (Figure 6a). To ensure that we would have sufficient KO mice, we used 4‐week‐old mice for the experiment. The surviving mice in the high‐fat diet group had significantly higher blood glucose levels and displayed more normal intestinal architecture, including increased villus length and mucous secretion (Figures 6b and S7a–c). We found distinct differences in bacterial diversity between the KO and KOHD groups and many similarities between the WTHD and KOHD groups (Figure 6c). The similarity of gut microbiota profiles in KO and WT mice under a high‐fat diet suggested that a high‐fat diet could improve and rebalance the gut dysbiosis in KO mice to a relatively normal state. Gut dysbiosis was naturally improved by the high‐fat diet intervention, as measured by the reduced abundance of harmful bacterial taxa, particularly Enterobacteriaceae and *E. coli* (Figures 6d,e, and S7d). Additionally, the reduced prevalence of *Bacteroides*, Proteobacteria, and *Enterococcus* and the enriched abundance of Actinobacteria, *Streptococcus*, and *Faecalibacterium* demonstrated the attenuation of gut dysbiosis (Figure 6d). Moreover, we found that the Enterobacteriaceae translocation to visceral organs was almost completely reversed by the high‐fat diet in KO mice (Figure 6f).

To confirm that the mechanism by which a high‐fat diet was able to attenuate premature aging was through rebalancing gut dysbiosis, we performed FMT with KOHD microbiota in WT mice. FMT effectiveness was evaluated by comparing the gut microbiota profiles of donors and recipients. This analysis showed that transplanted WT mice acquired KOHD microbiota (Figure S8a). We found that, compared with KO‐WT mice, the intestinal environment of KOHD‐WT mice was balanced, with a healthier intestinal tissue structure (Figures S8b,c) and a lower abundance of *E. coli* (Figure S8d). Moreover, we performed FMT from KOHD mice to 19‐day‐old KO mice (Figure 6g). In addition to the decreased relative abundance of *E. coli* in feces from KOHD‐KO mice compared to KO mice (Figure 6h), we also found that KOHD‐KO mice presented with decreased expression of inflammatory factors TNFα, IL‐1β, and IL‐6, and senescence markers p16 and p21 (Figure 6i). These data provide direct evidence of causality to claim that the effect of a high‐fat diet is mediated by a rebalancing of the gut microbiota.

Collectively, these data support the concept that a high‐fat diet restructures the balanced gut microbiota and specifically reduces the overgrowth of *E. coli*, ameliorating the progeria phenotype.

## 4 DISCUSSION

**FIGURE 7:**
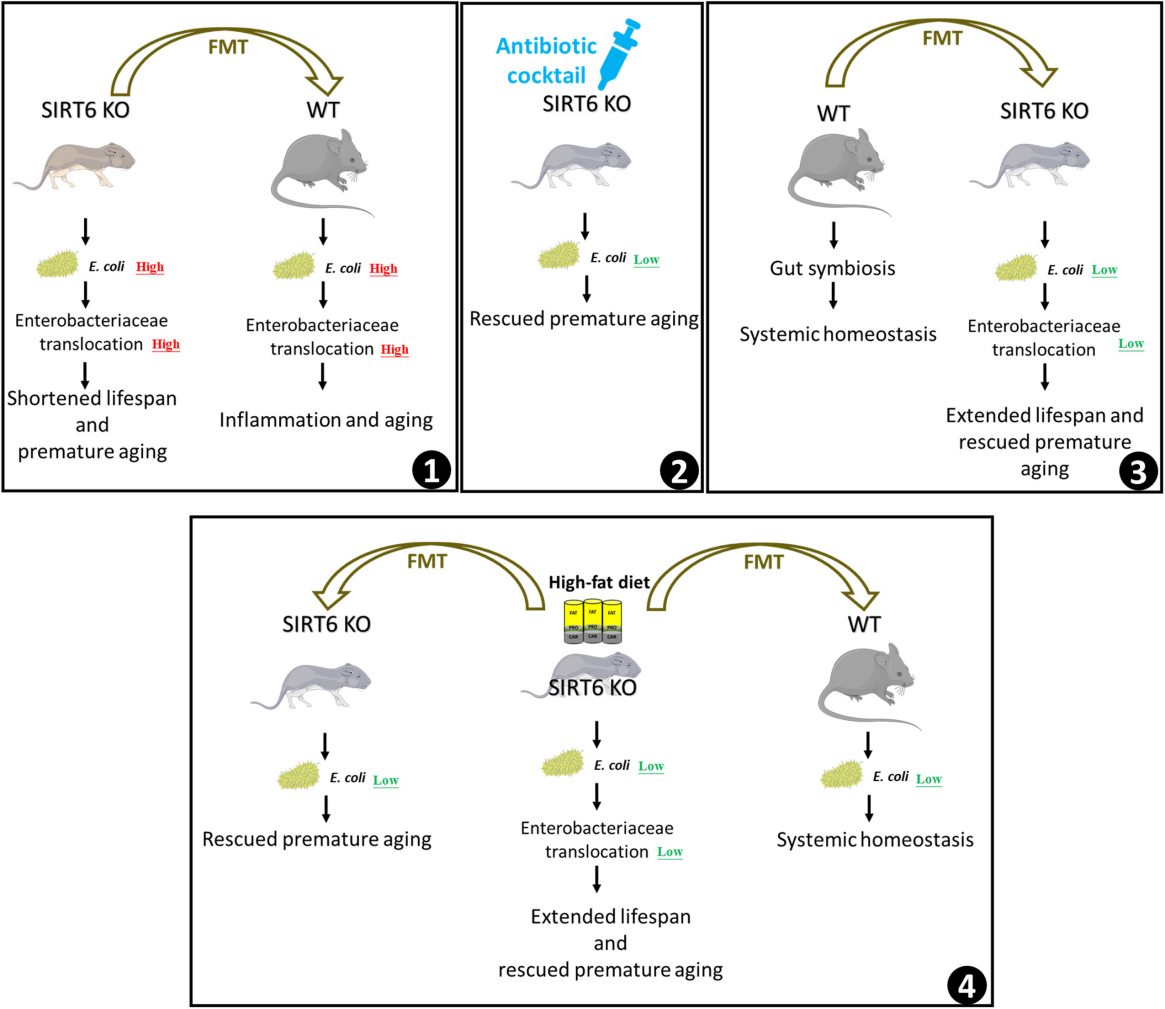
Working model for how gut microbiota remodeling mediates the alleviating of premature aging by a high‐fat diet. In SIRT6 KO mice, a high abundance of *E. coli* in the gut aggravates Enterobacteriaceae translocation to visceral organs, contributing to shortened lifespan and premature aging. Transferring the microbiome of KO to WT mice confers premature aging phenotypes due to the overgrowth of *E. coli* and increased Enterobacteriaceae translocation (Box 1). Antibiotic treatment reduces the abundance of *E. coli* in KO mice, attenuating inflammation and cell senescence of KO mice (Box 2). Improving gut dysbiosis by FMT from WT mice decreases the *E. coli* abundance and Enterobacteriaceae translocation in KO mice, resulting in extended lifespan and rescued premature aging (Box 3). A high‐fat diet extends the lifespan and rescues the premature aging of KO mice by the same mechanism. KO mice transplanted with microbiota from KOHD mice also have a low level of *E. coli* and rescued premature aging phenotypes. WT mice transplanted with microbiota from KOHD mice show systemic homeostasis (Box 4)

High‐fat diets have been shown to enhance the lifespan and health span in both naturally aging and prematurely aging mice (Roberts et al., 2018; Scheibye‐Knudsen et al., 2014; Shi et al., 2021). However, there is a dearth of information on how the microbiome is altered by a high‐fat diet and the contribution of these changes to phenotypical aging improvements. Here, we found that SIRT6 KO mice, a useful aging research model, have an imbalanced microbiome signature and that this profile, in addition to its associated accelerated aging characteristics, could be transferred to WT mice through fecal transplantation from SIRT6 KO donors. Conversely, FMT from WT mice to SIRT6 KO mice rescued the shortened lifespan and gut dysbiosis observed in SIRT6 KO mice. The antibiotic treatment effectively inhibited the overgrowth of *E. coli* and rescued premature aging phenotypes in KO mice. We traced the mechanism of this gut dysbiosis‐induced aging to the overgrowth of *E. coli*, particularly STEC, which enhances Enterobacteriaceae translocation into visceral organs and the acceleration of certain aging phenotypes. Importantly, we demonstrated that a high‐fat diet could reestablish a healthy gut microbiome and reverse shortened lifespan; this occurred primarily through the reduction of *E. coli* abundance in the gut and Enterobacteriaceae translocation to visceral organs (Figure 7). Our findings highlight the potential of a novel therapeutic strategy to treat aging and aging‐associated diseases by targeting the link between the gut microbiome and the aging process.

A marked increase in *E. coli* abundance was observed in SIRT6 KO mice compared to WT mice. This led us to wonder how a global deficiency in SIRT6 could drive the growth and colonization of *E. coli* in the intestine. SIRT6 inhibition results in decreased differentiation and immunostimulatory capacity of human dendritic cells (DCs), reducing their antigen uptake, processing, and presentation. Disabled DCs cannot efficiently activate T‐ and B‐cell immune responses, allowing pathogenic bacterial populations to overwhelm the microbiome (Honda & Littman, 2016; Lasigliè et al., 2016). Therefore, aberrant immune system function potentially contributes to a suitable growth microenvironment for *E. coli* in SIRT6 KO mice (Small et al., 2013). As consistent with previous reports that no physiological effects are observed in SIRT6+/− mice without stress (Naiman et al., 2019; Wang et al., 2016), we did not find gut dysbiosis in 1‐month‐old SIRT6+/− mice under normal growth conditions, suggesting that SIRT6 haploinsufficiency retains sufficient activity. Overabundant *E. coli* has been widely observed in older adults (Bana & Cabreiro, 2019; Biagi et al., 2012). This overgrowth drives overall increases in Enterobacteriaceae family bacteria, which further negatively affects both the relative abundance of other probiotics and overall microbial diversity, supporting a link between increases in specific coliforms and decreased microbial diversity with aging (Leite et al., 2021). Therefore, an overabundance of both *E. coli* and Enterobacteriaceae are likely the initial drivers of dysbacteriosis symptoms in SIRT6 KO mice. Consistent with our findings that gut dysbiosis can drive system inflammation and visceral senescence, it has been reported that the overgrowth of gut microbes can result in increased bacterial translocation and tissue damage (Nicolle et al., 2004). Our data suggest that Enterobacteriaceae translocation, driven by overabundant *E. coli*, leads to senescence of spleen, kidney, and liver tissues. This may be the causal link between gut dysbiosis and aging.

The gut microbiome is highly sensitive to dietary influences and plays a central role in coordinating the host metabolism (Ang et al., 2020). In our study, a short‐term high‐fat diet significantly decreased *E. coli* abundance and improved gut dysbiosis, contributing to lifespan extension and reversal of premature aging in SIRT6 KO mice. When mice were switched to a fat‐rich diet, the gut microbiota composition was altered within 24 h, driven by an increase in dietary fat content rather than the obese state (Hildebrandt et al., 2009). A high‐fat diet enhances bile secretion to facilitate lipid digestion (Islam et al., 2011). The concentration of total bile acids, including secondary bile acids such as deoxycholic acid and lithocholic acid, increases during the consumption of high‐fat diets compared to high‐carbohydrate diets in humans and animal models (Reddy, 1981). Bárcena found that in vivo supplementation of cholic acid in the diet facilitates beneficial effects for progeria, including improved overall health and enhanced median and maximal survival, suggesting that the modulation of bile acid metabolism could regulate longevity in mice (Bárcena et al., 2018). Chenodeoxycholic acid, ursodeoxycholic acid, and lithocholic acid have potent antibacterial activities against the populations of *E. coli* (Kong et al., 2011; Wang et al., 2019). Mechanistically, the effects of deoxycholate on *E. coli* include increases in the lag time constant and generation time and decreases in the growth rate constant and total cell yield of this microorganism. Therefore, one explanation for the inhibited growth of *E. coli* that is induced by a high‐fat diet may be increased bile acid secretion. Another potential mechanism of a high‐fat diet in dysbiosis amelioration is the effect of the coconut oil used in our study. Medium‐chain saturated fatty acids such as caprylic acid, a characteristic nutrient in coconut oil, have shown significant antimicrobial activity against *E. coli* (Lemarié et al., 2018; Marounek et al., 2003). Moreover, the alteration in the abundance of other pathogens such as Proteobacteria possibly results from decreases in the family Enterobacteriaceae, especially the genus *Escherichia* (Leite et al., 2021). We hope to address further functions of bile acids in health maintenance and disease defense in future studies.

High‐fat diets sometimes referred to as high‐calorie diets have been associated with calorie overconsumption in most studies (Bisanz et al., 2019). Western diets, which tend to be high in fat and/or calories, are associated with an increased incidence of metabolic syndromes, malignancies, and other age‐related dysfunctions (Albenberg & Wu, 2014). In contrast, high‐fat, low‐carbohydrate diets with decreased energy intake result in fat mass reduction and weight loss in humans, accompanied by reduced circulating triglycerides and insulin resistance (Veum et al., 2017). Additionally, isocaloric high‐fat diets enriched in medium‐chain triglycerides ameliorate metabolic aberrations associated with obesity, reduce total body fat, improve overall metabolic health, and induce thermogenesis in the liver and subcutaneous white adipose tissues (Rial et al., 2020). Recent research reports that an isocaloric, moderately high‐fat diet, with 34.6% energy from fat, extends the lifespan in male rats and *Drosophila* (Shi et al., 2021). Coconut oil is anti‐obesogenic, anti‐inflammatory, and insulin‐sensitizing, in contrast to soybean oil, which is obesogenic and diabetogenic, and is associated with unfavorable changes in the gut microbiota (Deol et al., 2015; Wan et al., 2019). In our study, both WT and SIRT6‐KO mice were fed an isocaloric coconut oil‐based high‐fat diet. No obvious gut dysbiosis or metabolic disorder was detected in the WT mice after 1 week of high‐fat diet treatment. Therefore, the long‐term effects of an isocaloric high‐fat diet on the gut microbiota, metabolism, and lifespan in young and aging individuals warrant further research.

In summary, our findings highlight the potential of a novel therapeutic strategy to treat aging and age‐associated diseases by targeting the link between the gut microbiome and the aging process. Continued progress in dissecting the mechanistic basis of these observations will be fundamental to providing more personalized approaches in the employment of dietary interventions to treat aging and aging‐related diseases.

## AUTHOR CONTRIBUTIONS

K.X., Z.C.L., and Z.W. conceived the study; K.X., Z.C.L., and Z.W. designed the experiments; K.X. and Y.N.G. conducted most of the experiments and data analyses; Y.D.W., K.Z.P., and L.P. performed animal feeding, dissection, and tissue staining. S.C. and V.L. contributed to data analyses and interpretation. Y.R. and Y.Q. conducted staining analyses and quantification; X.L., Y.Q., Q.F.L., and Z.W. contributed to the discussion and data interpretation; K.X., Z.C.L., V. L., and Z.W. wrote the manuscript. All authors read and approved the final manuscript.

## CONFLICT OF INTEREST

The authors declare no competing interests.

## Supporting information


Appendix S1

## Data Availability

The accession number for the 16S gut microbiome data reported in this paper is NCBI: PRJNA629383 and PRJNA757209. All other data and code used to analyze data are available upon reasonable request.
