# Supplementary material for: Decreased Enterobacteriaceae translocation due to gut microbiota remodeling mediates the alleviation of premature aging by a high‐fat diet
Source: Aging Cell. 2022 Dec 25;22(2):e13760. doi: 10.1111/acel.13760 (PMC9924944; doi:10.1111/acel.13760)
Supplement: Supplementary file 1 — Appendix S1 [file ACEL-22-e13760-s001.docx]

**
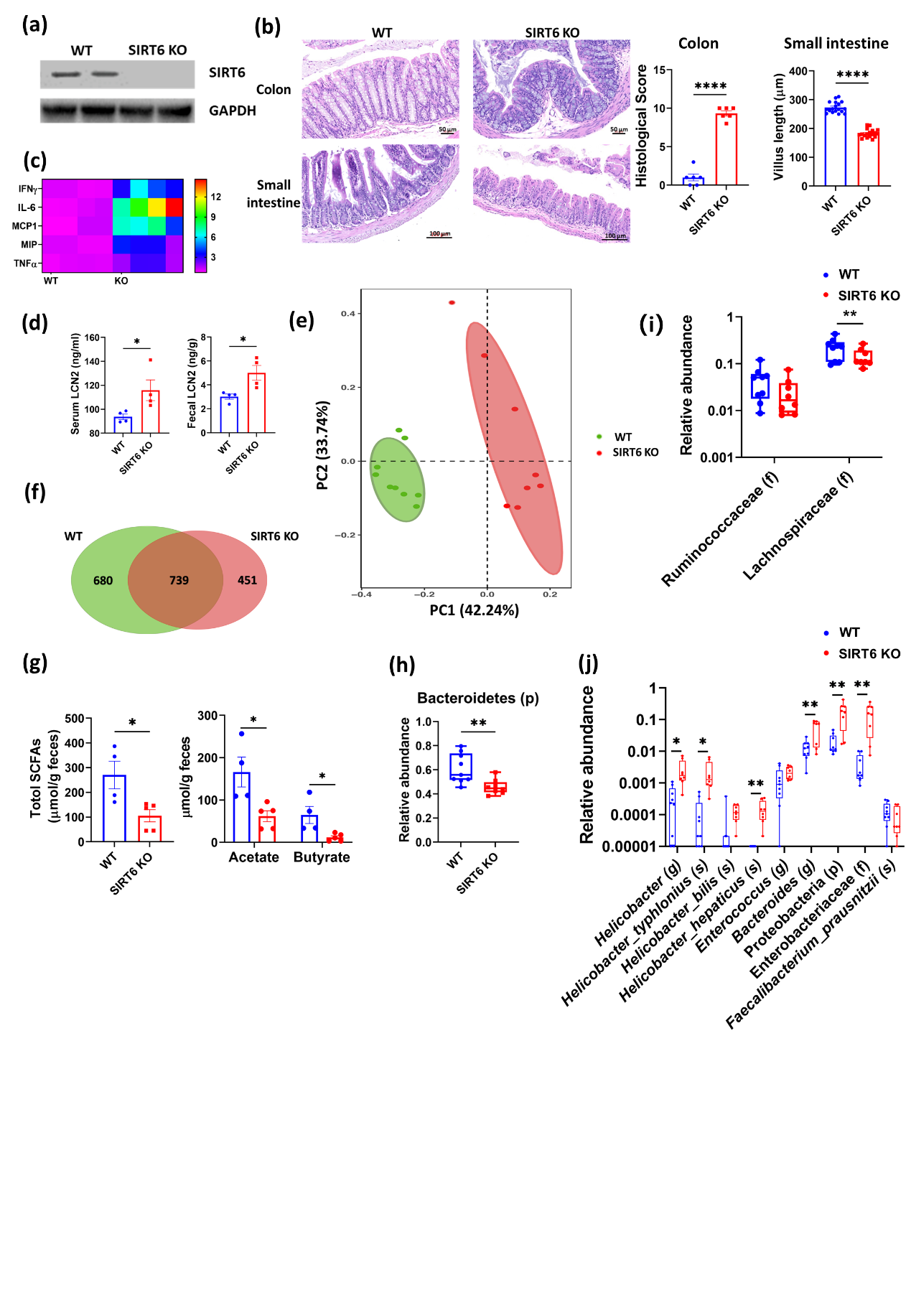
Figure S1. The gut microbiome is altered in SIRT6 knockout mice.**

(a) The expression of SIRT6 in the colon was tested by western blotting, and GAPDH served as an internal reference. (n≥3 biological replicates with representative example shown.)

(b) Representative images of H&E-stained colon and small intestine tissue sections. Histological score and quantification of villus length were shown (n=6 per group). For colon histological score, each dot represents an individual mouse. For villus length, three fields of each section were randomly selected, and then all villi in this field were measured and the average villi length of each field was calculated, and each dot represents a field.

(c) Heatmap revealing relative mRNA expression levels of IFNγ, IL-6, MCP1, MIP, and TNFα in colon tissue (n=4 mice per group).

(d) Serum and feces inflammatory maker LCN2 concentrations were tested (n=4 mice per group).

(e) Principal Coordinates Analysis (PCoA) plot of unweighted UniFrac distance was carried out on even operational taxonomic units (OTUs) table data (*p*=0.014; PERMANOVA) between SIRT6 knockout and WT groups of 4-week-old mice. Each dot represents an individual mouse (WT, n=9; SIRT6 KO, n=8).

(f) Venn diagram shows the number of shared and unique OTUs detected in WT and SIRT6 KO mice.

(g) The concentration of total SCFAs and acetate and butyrate in fecal content (WT, n=4; SIRT6 KO, n=5).

(h) Relative abundance of Bacteroidetes (WT, n=9; SIRT6 KO, n=8).

(i) Relative abundance of SCFA-producing taxa (WT, n=9; SIRT6 KO, n=8).

(j) Depiction of bacterial taxa whose relative abundance was different between WT and SIRT6 KO mice (WT, n=9; SIRT6 KO, n=8).

**p*<0.05, ***p*<0.01, *****p*<0.0001

**
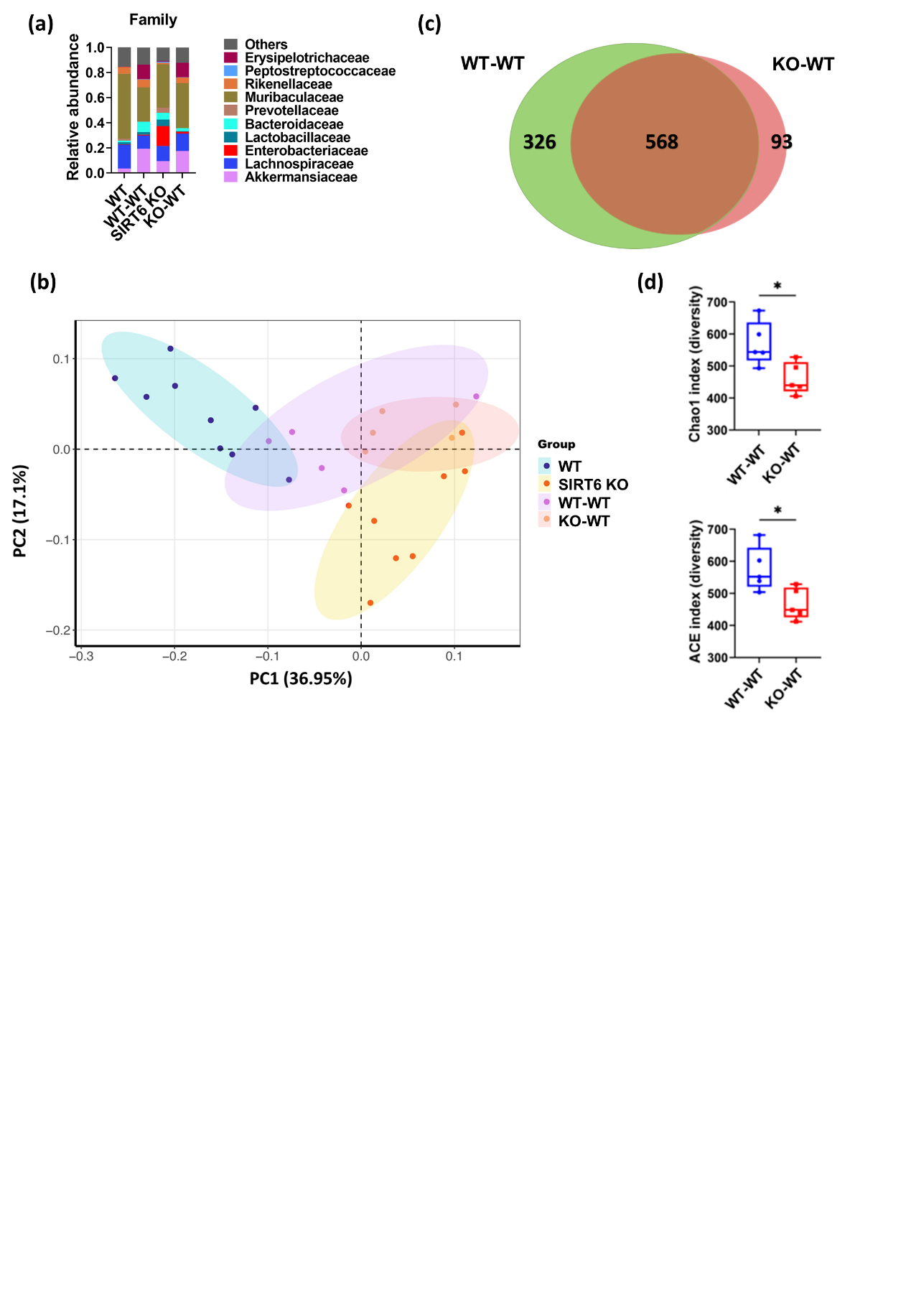
**

**Figure S2. Gut microbiota from SIRT6 KO mice induces gut dysbiosis in WT mice.**

(a) Average relative abundance of the top 10 abundant bacteria at the family level in the FMT experiment, comparing the donor mice with the recipient mice (WT, n=9; WT-WT, n=5; SIRT6 KO, n=8; KO-WT, n=5).

(b) Principal Coordinates Analysis (PCoA) plot of weighted UniFrac distance. Each dot represents an individual mouse (WT, n=9; WT-WT, n=5; SIRT6 KO, n=8; KO-WT, n=5).

(c) Venn diagram shows the number of shared and unique operational taxonomic units (OTUs) detected in WT-WT and KO-WT mice.

(d) Alpha diversity between WT-WT and KO-WT mice was analyzed based on the Chao1 index and ACE index (n=5 per group).

**p*<0.05

**
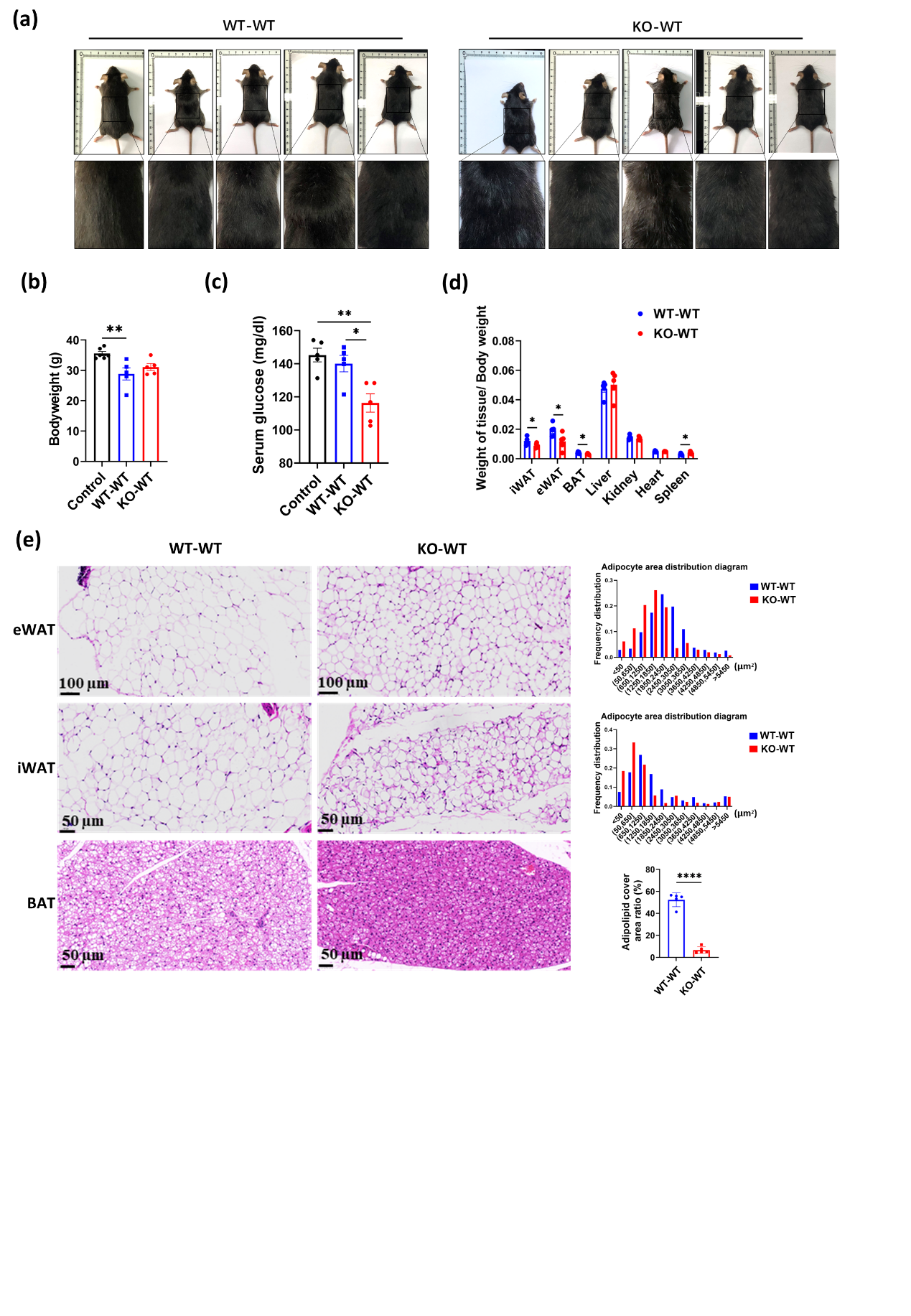
**

**Figure S3. Accelerated aging is observed in WT mice transplanted with gut microbiota from SIRT6 KO mice.**

(a) Replicates of hair conditions in Figure 1f of WT-WT mice and KO-WT mice.

(b-c) Bodyweight and serum glucose level of Control mice (un-transplanted mice), WT-WT mice and KO-WT mice (n=5-6 per group).

(d) Different groups of mice were sacrificed, and then tissues and organs were dissected and weighted (n=5 per group).

(e) Representative image of H&E staining of eWAT (Epididymis white adipocyte tissue), iWAT (Inguinal white adipocyte tissue), and BAT (Brown adipocyte tissue). Adipocyte distribution in eWAT and iWAT, and adipolipid cover area ratios were measured (n=5 per group). Each dot represents an individual mouse.

**p*<0.05, ***p*<0.01, *****p*<0.0001

**
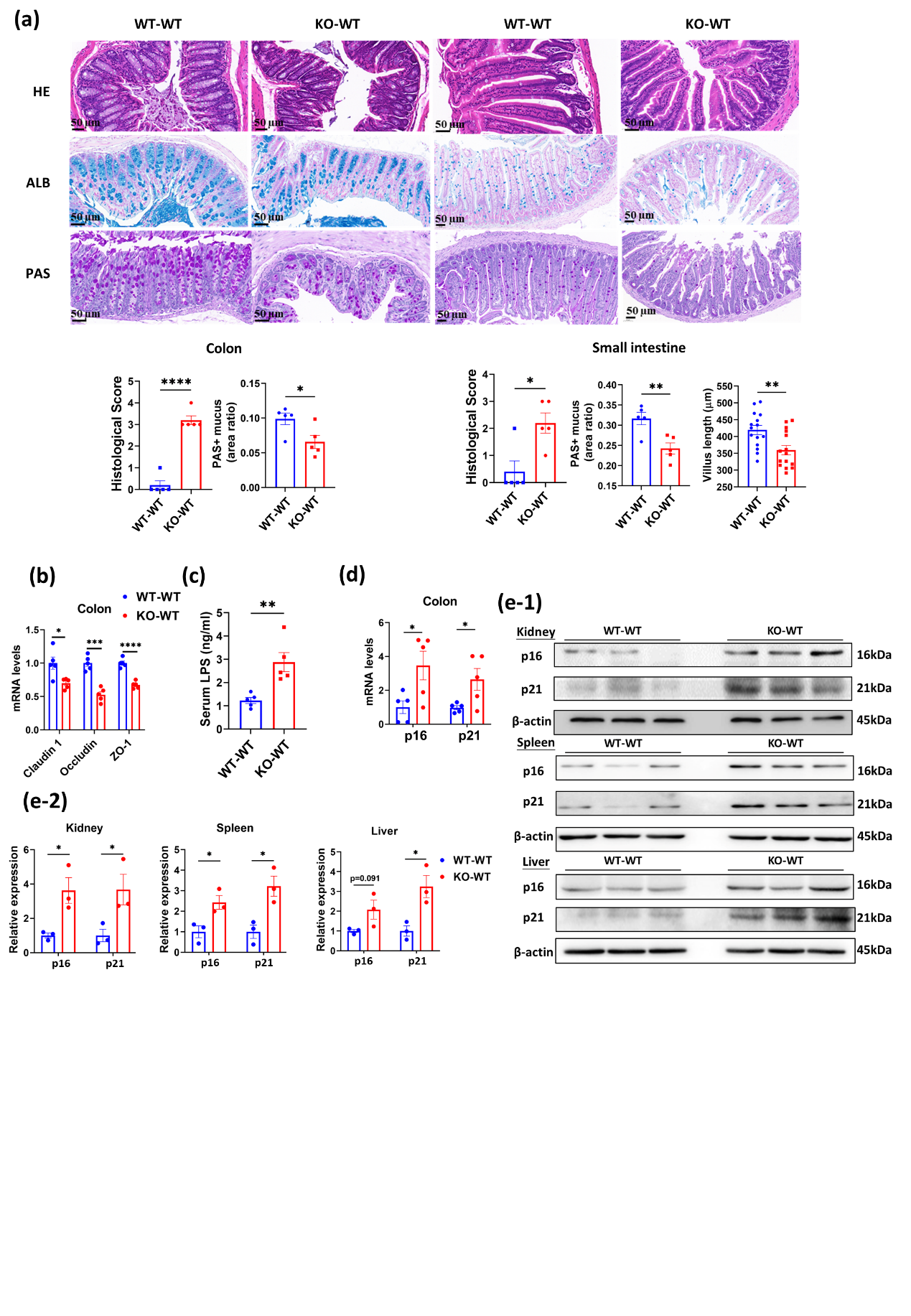
Figure S4. FMT of SIRT6 KO donor microbiomes into WT recipients results in inflammation and cell senescence.**

(a) Representative sections of colon and small intestine stained with H&E (HE), Alcian blue (ALB), and Periodic acid-Schiff (PAS). Histological score, villus length, and PAS^+^ mucus was quantified individually (n=5 per group). Each dot represents an individual mouse. For villus length, three fields of each section were randomly selected, and then all villi in this field were measured and the average villi length of each field were calculated, and each dot represents a field.

(b) The relative mRNA levels of tight junction proteins including Claudin 1, Occludin, and ZO-1 measured by qPCR in colon tissue (n=5 per group).

(c) Serum LPS concentration (n=5 per group).

(d) The mRNA levels of p21 and p16 were measured by qPCR in colon tissue (n=5 per group).

(e) Expression of p16 and p21 measured by western blot in kidney, spleen, and liver. Each dot represents an individual mouse in the statistical graph. (n=3 per group).

**p*<0.05, ***p*<0.01, ****p*<0.001, *****p* <0.0001

**
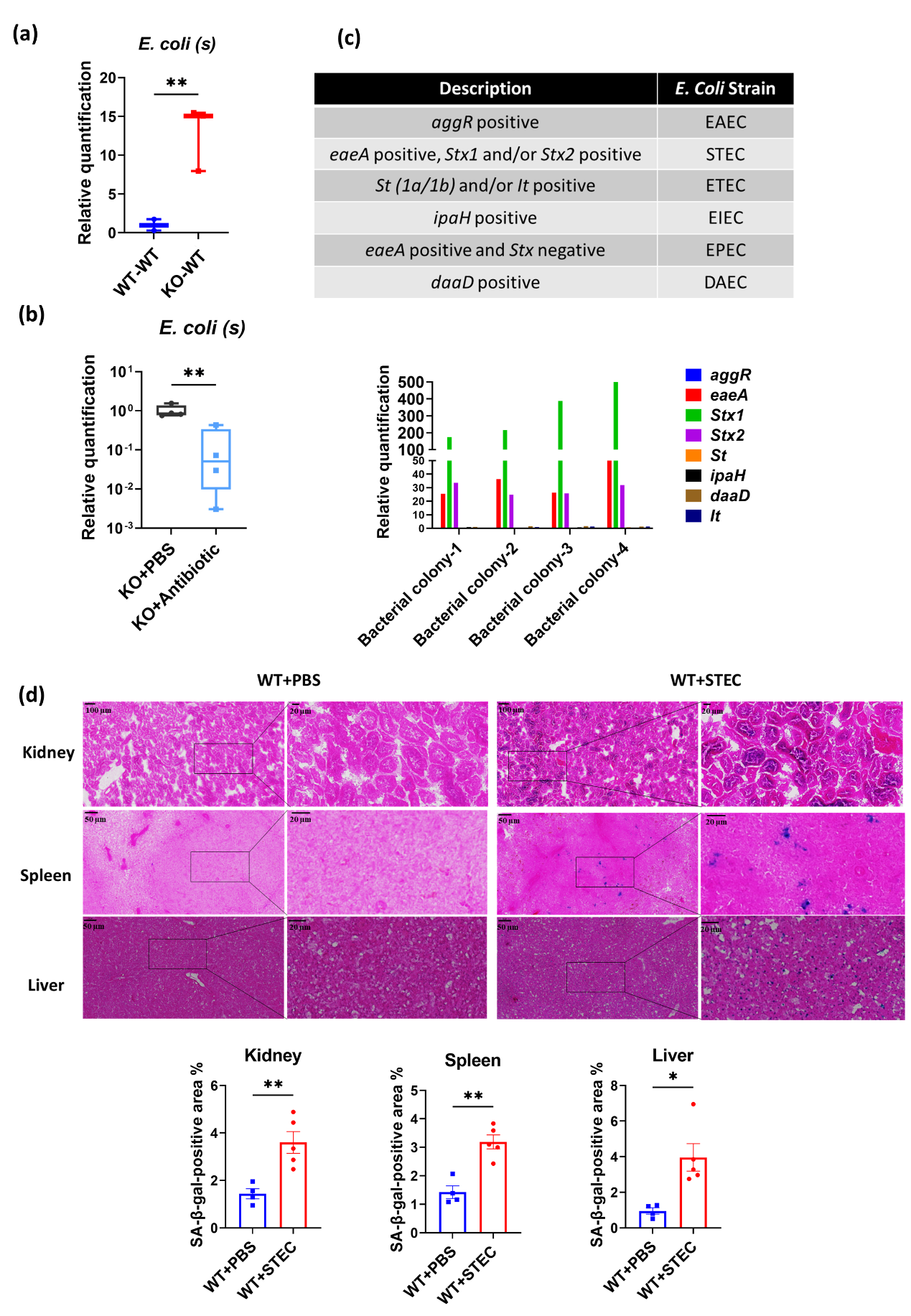
**

**Figure S5. STEC strain is validated in KO mice.**

(a) Validation of the differences in the abundance of *E. coli* between WT-WT and KO-WT mice 2 months after FMT by qPCR (n=3 per group).

(b) Validation of the differences in abundance of *E. coli* between KO+PBS and KO+Antibiotic mice by qPCR (n=4 per group).

(c) Relative expression of genes for the identification of different *E. coli* strains. Positive for *eaeA, Stx1* and/or *Stx2* indicate the STEC.

(d) Representative images and quantitative analysis of SA-β-gal activity assay in kidney, spleen and liver (WT+PBS, n=4; WT+STEC, n=5).

**p*<0.05, ***p*<0.01

**
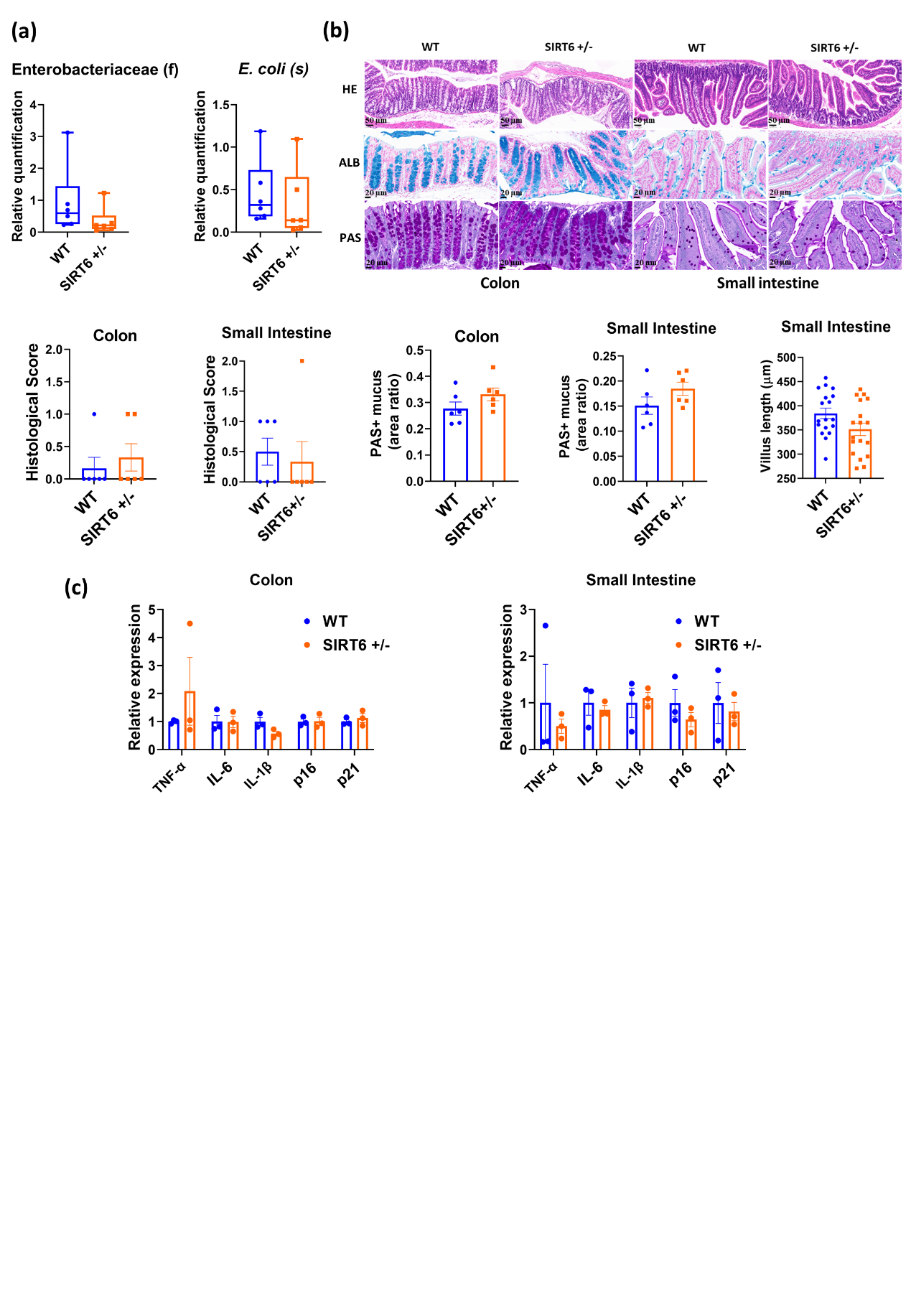
Figure S6. Effects of SIRT6 heterozygous on gut microbiota.**

(a) Validation of the differences in abundance of Enterobacteriaceae and *E. coli* between WT and SIRT6 +/- mice by qPCR (n=6 per group).

(b) Representative sections of colon and small intestine stained with H&E (HE), Alcian blue (ALB), and Periodic acid-Schiff (PAS). Histological score, villus length, and PAS^+^ mucus was quantified individually (n=6 per group). Each dot represents an individual mouse. For villus length, three fields of each section were randomly selected, and then all villi in this field were measured and the average villi length of each field were calculated, and each dot represents a field.

(c) Relative mRNA expression of inflammatory factors including TNFα, IL-1β and IL-6, and senescence markers including p21 and p16 in colon and small intestine from WT and SIRT6+/- mice were measured by qPCR (n=3 per group).


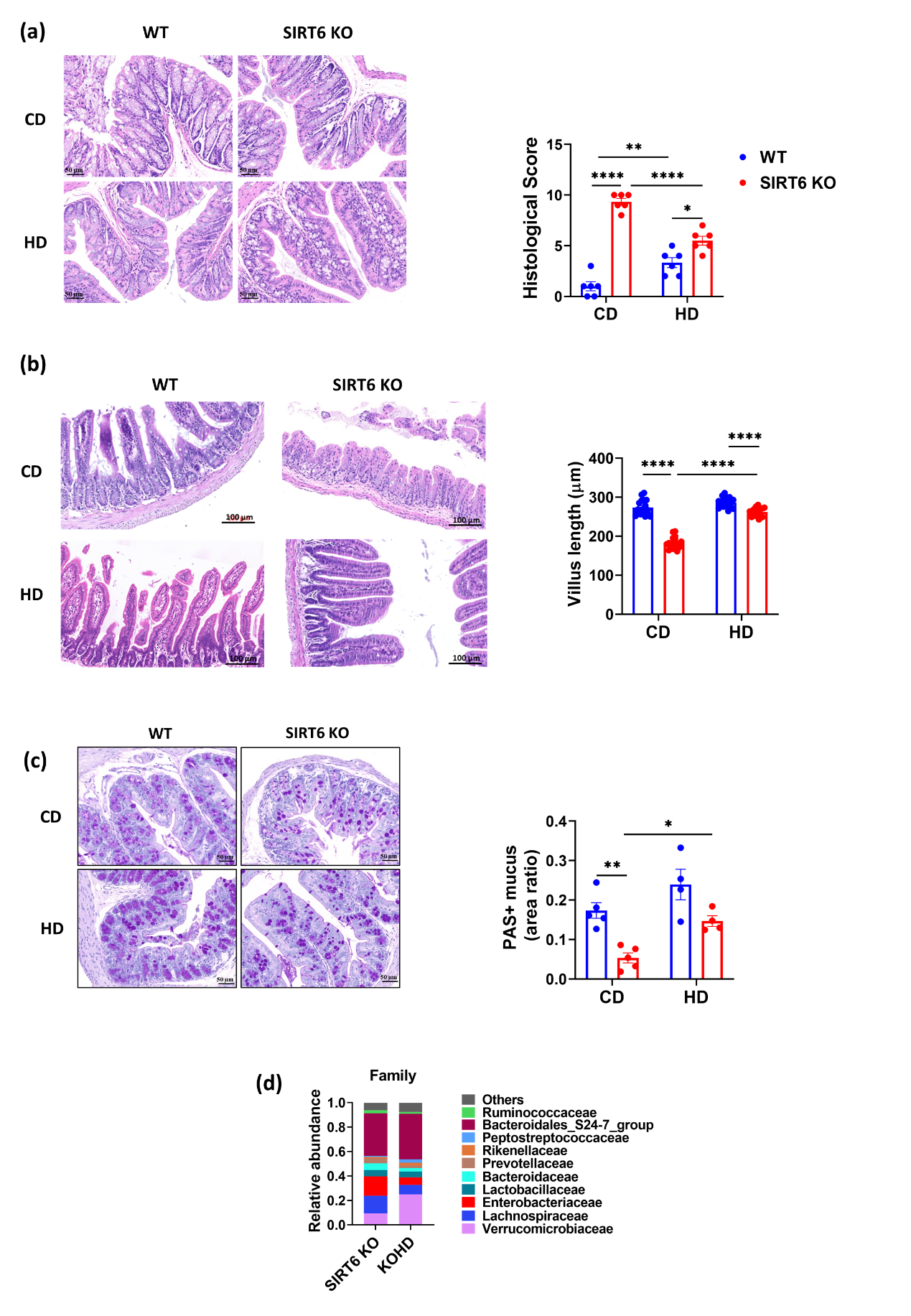


**Figure S7. Effects of high-fat diet on SIRT6 knockout mice.**

(a-b) Representative images of H&E staining colon and small intestine sections from WT, SIRT6 KO, WTHD, and KOHD group. Histological score of colon and villus length was quantified (n=6 per group). For colon histological score, each dot represents an individual mouse. For villus length, three fields of each section were randomly selected, and then all villi in this field were measured and the average villi length of each field was calculated, and each dot represents a field.

(c) Representative PAS staining image and its quantification of colon (n=4-5 per group). Each dot represents an individual mouse.

(d) Average relative abundance of the top 10 abundant bacteria at the family level among SIRT6 KO and KOHD mice (SIRT6 KO, n=8; KOHD, n=8).

**p*<0.05, ***p*<0.01, *****p*<0.0001


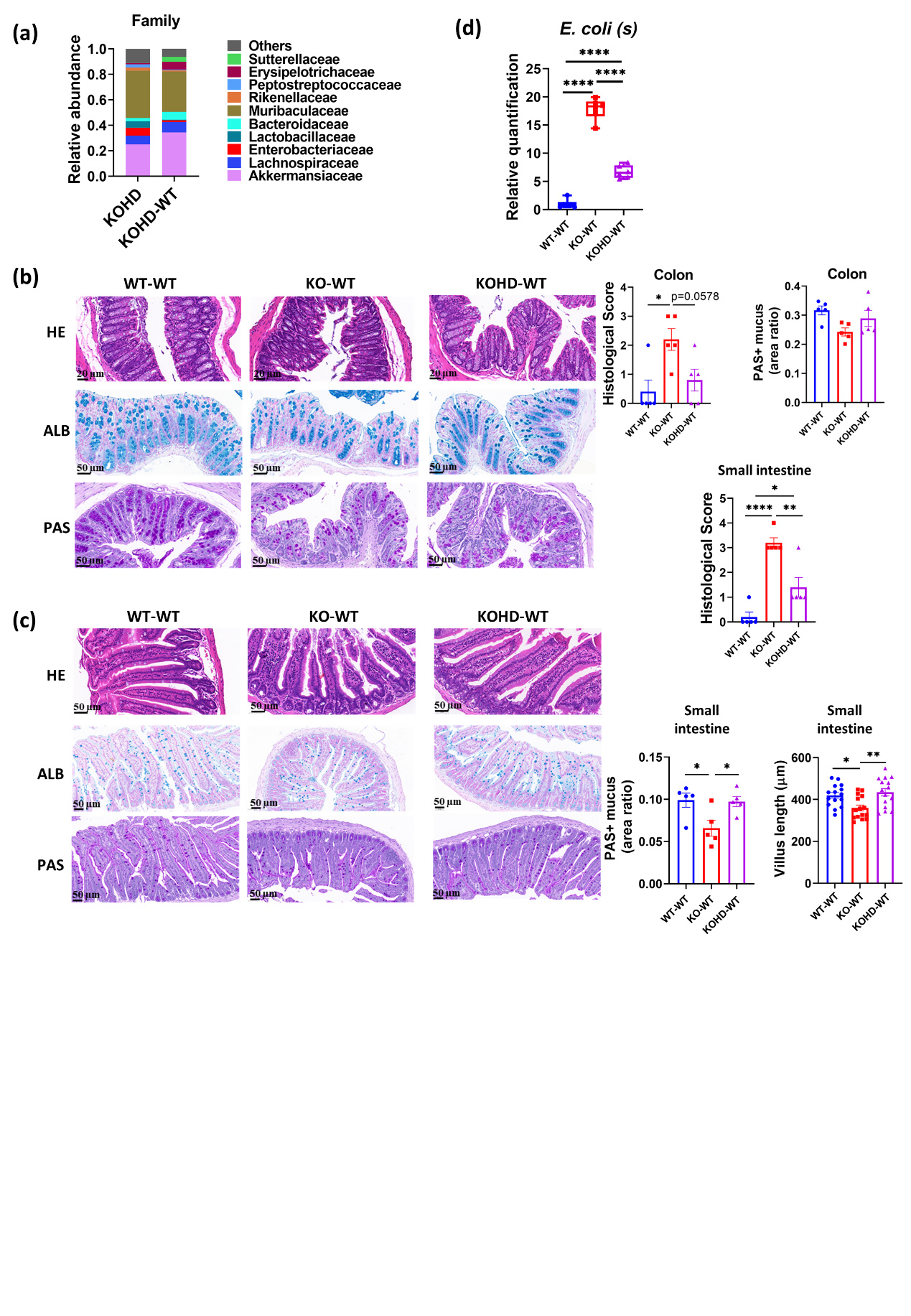


**Figure S8. Effects of FMT with KOHD mice on WT recipients.**

(a) Average relative abundance of the top 10 abundant bacteria at the family level in the FMT experiment, comparing the donor mice and recipient mice (KOHD, n=8; KOHD-WT, n=5).

(b-c) Representative sections of the colon (b) and small intestine (c) stained with H&E (HE), Alcian blue (ALB), and Periodic acid-Schiff (PAS). Histological score, villus length, and PAS^+^ mucus were quantified individually (n=5 per group). Each dot represents an individual mouse in most of the histograms, except villus length statistics. For villus length, three fields of each section were randomly selected, and then all villi in this field were measured and the average villi length of each field was calculated, and each dot represents a field.

(d) Validation of the differences in the abundance of *E. coli* among WT-WT, KO-WT, and KOHD-WT mice by qPCR (n=6 per group).

**p*<0.05, ***p*<0.01, *****p*<0.0001

**Tables**

| **Mouse** | **Bodyweight (g)** | **Weight of organ (g)** | | | | | | |
| --- | --- | --- | --- | --- | --- | --- | --- | --- |
|  |  | **iWAT** | **eWAT** | **BAT** | **Liver** | **Kidney** | **Heart** | **Spleen** |
| WT-WT-1 | 33.74 | 0.529 | 0.865 | 0.150 | 1.291 | 0.448 | 0.184 | 0.100 |
| WT-WT-2 | 31.17 | 0.331 | 0.615 | 0.118 | 1.568 | 0.395 | 0.141 | 0.068 |
| WT-WT-3 | 28.05 | 0.324 | 0.562 | 0.133 | 1.445 | 0.400 | 0.127 | 0.078 |
| WT-WT-4 | 21.86 | 0.087 | 0.095 | 0.073 | 1.044 | 0.316 | 0.111 | 0.083 |
| WT-WT-5 | 29.33 | 0.209 | 0.442 | 0.106 | 1.453 | 0.490 | 0.141 | 0.097 |
| KO-WT-1 | 29.00 | 0.315 | 0.543 | 0.106 | 1.043 | 0.411 | 0.140 | 0.089 |
| KO-WT-2 | 31.08 | 0.223 | 0.403 | 0.079 | 1.649 | 0.396 | 0.152 | 0.110 |
| KO-WT-3 | 35.08 | 0.301 | 0.328 | 0.112 | 1.977 | 0.479 | 0.175 | 0.178 |
| KO-WT-4 | 31.68 | 0.238 | 0.432 | 0.098 | 1.840 | 0.482 | 0.147 | 0.133 |
| KO-WT-5 | 28.73 | 0.282 | 0.111 | 0.088 | 1.378 | 0.360 | 0.134 | 0.077 |

Table S1. The weight of the whole body and different organs and tissues between WT-WT and KO-WT mice.

iWAT (Inguinal white adipocyte tissue), eWAT (Epididymis white adipocyte tissue), and BAT (Brown adipocyte tissue)

| Based on AIN-93G w/65% kcal% from Coconut Oil | | | | |
| --- | --- | --- | --- | --- |
| Product | SD17082401 | | AIN-93G | |
|  | gm% | kcal% | gm% | kcal% |
| Protein | 26.1 | 19 | 21 | 19 |
| Carbohydrate | 22.4 | 16 | 63 | 64 |
| Fat | 39.7 | 65 | 7.5 | 17 |
| Total |  | 100 |  | 100 |
| kcal/gm | 5.5 |  | 3.9 |  |
| Ingredient | gm | kcal | gm | kcal |
| Casein, 30 Mesh | 187 | 748 | 210 | 739.2 |
| L-Cystine | 3 | 12 | 3 | 12 |
| Corn Starch |  |  | 382.5 | 1530 |
| Sucrose | 27.5 | 110 | 100 | 400 |
| Cellulose, BW200 | 50 | 0 | 50 | 0 |
| Maltodextrin | 122.5 | 490 | 132 | 528 |
| Coconut Oil | 289 | 2601 | 0 | 0 |
| Soybean Oil |  |  | 75 | 675 |
| Mineral Mix S10022G | 35 | 0 | 35 | 0 |
| Vitamin Mix V10037 | 10 | 40 | 10 | 40 |
| Choline Bitartrate | 2.5 | 0 | 2.5 | 0 |
| t-Butylhydroquinone | 0 | 0 | 0.014 | 0 |
| Total | 727 | 4001 | 1000.014 | 3924.2 |

Table S2. Composition of the diets in this study.

| **Gene** | **Forward** | **Reverse** |
| --- | --- | --- |
| *IFNγ* | CAGCAACAGCAAGGCGAAAAAGG | TTTCCGCTTCCTGAGGCTGGAT |
| *IL-6* | TACCACTTCACAAGTCGGAGGC | CTGCAAGTGCATCATCGTTGTTC |
| *MCP1* | GCCTCAAACCTTCCAAATCA | GTGAAGTCGGCCAAAGTTGT |
| *TNFα* | TCTTCTCATTCCTGCTTGTGG | GGTCTGGGCCATAGAACTGA |
| *IL-1β* | TGGACCTTCCAGGATGAGGACA | GTTCATCTCGGAGCCTGTAGTG |
| *p16* | TGTTGAGGCTAGAGAGGATCTTG | CGAATCTGCACCGTAGTTGAGC |
| *p21* | TCGCTGTCTTGCACTCTGGTGT | CCAATCTGCGCTTGGAGTGATAG |
| *Claudin1* | GGACTGTGGATGTCCTGCGTTT | GCCAATTACCATCAAGGCTCGG |
| *Occludin* | TGGCAAGCGATCATACCCAGAG | CTGCCTGAAGTCATCCACACTC |
| *ZO-1* | GTTGGTACGGTGCCCTGAAAGA | GCTGACAGGTAGGACAGACGAT |
| *GAPDH* | CATCACTGCCACCCAGAAGACTG | ATGCCAGTGAGCTTCCCGTTCAG |
| *β-actin* | CATCCGTAAAGACCTCTATGCCAAC | ATGGAGCCACCGATCCACA |
| *E. coli* | GTTAATACCTTTGCTCATTGA | ACCAGGGTATCTAATCCTGTT |
| *Enterobacteriaceae* | CATTGACGTTACCCGCAGAAGAAGC | CTCTACGAGACTCAAGCTTGC |
| *eaeA* | ATGCTTAGTGCTGGTTTAGG | GCCTTCATCATTTCGCTTTC |
| *aggR* | CGAAAAAGAGATTATAAAAATTAAC | GCTTCCTTCTTTTGTGTAT |
| *daaD* | TGAACGGGAGTATAAGGAAGATG | GTCCGCCATCACATCAAAA |
| *Stx1* | CTGGATTTAATGTCGCATAGTG | AGAACGCCCACTGAGATCATC |
| *Stx2* | GGCACTGTCTGAAACTGCTCC | TCGCCAGTTATCTGACATTCTG |
| *It* | TCTCTATGTGCATACGGAGC | CCATACTGATTGCCGCAAT |
| *IpaH* | GTTCCTTGACCGCCTTTCCGATACCGTC | GCCGGTCAGCCACCCTCTGAGAGTAC |
| *St* | TTTCCCCTCTTTTAGTCAGTCAA  TGCTAAACCAGTAGAGTCTTCAAAA | GCAGGATTACAACACAATTCACAGCAG |
| Universal Eubacteria | ACTCCTACGGGAGGCAGCAGT | GTATTACCGCGGCTGCTGGCAC |
|  |  |  |

Table S3. The primers for qPCR.

**Methods**

**Mouse generation and study**

SIRT6^tm1.1Cxd^ mice of a 129S6/SvEvTac (129Sv) background were purchased from the Jackson Laboratory (Bar Harbor, ME, USA). Male 129Sv SIRT6 ^tm1.1Cxd^ mice were mated with C57BL/6J CMV-Cre female mice (Nanjing Biomedical Research Institute) to generate F1 heterozygous mice. F1 heterozygous mice repeatedly backcrossed to 129Sv WT mice for more than 10 generations, 99.9% of its genetic composition will be the genetic background of 129Sv. And then, 129Sv SIRT6 heterozygous (SIRT6+/-) mice were interbred to generate WT, SIRT6 KO and SIRT6+/- mice. The mice were housed at the Laboratory Animal Research Center, Tsinghua University. All mouse experiments were performed on males, complied with regulations and ethics guidelines, and were approved by the International Animal Care and Use Committee (IACUC) of Tsinghua University. Heterozygous breeding produced progeny with the expected Mendelian ratios and heterozygous mice were used as breeders for our studies. Animal rooms were maintained at 23 °C with a 12-h light/dark cycle. Mice were identified by genotyping. After tail or toe clipping, tissues were digested for 3 h at 55 ℃ in 500 μL of 1 M Tris-HCl, 5 M NaCl, 0.5 M EDTA, and 10% SDS. Protease K and RNase A were also added to the lysis buffer. Isopropanol and ethyl alcohol were used for DNA extraction. Then, the following primers were used for PCR: forward: 5′-AGTGAGGGGCTAATGGGAAC-3′; reverse: 5′-CTGACGGTGTCTTCACAAACTCAC-3′. If the PCR product was 600 bp long, we considered the genotype to be SIRT6 knockout whereas 2000 bp indicated WT.

SIRT6 knockout (SIRT6 KO, 129Sv) mice, wild type (WT, 129Sv) and SIRT6 heterozygous (SIRT6 +/-, 129Sv) littermates were individually caged after weaning with a plaything to allow for acclimation to the animal facility. Fecal samples were obtained under sterile conditions, stored in a sterile 1.5 mL centrifuge tube at -80 °C until microbiome profiling analysis. Mice were sacrificed with carbon dioxide and blood collection was performed immediately via cardiac puncture. Serum was obtained after centrifugation at 1500 g for 15 min at 4 °C and was frozen at -80 °C until thawing for an assay. Then, the colon, intestine, and other tissues were removed and divided separately. Tissues prepared for section staining were post-fixed in phosphate-buffered 4% paraformaldehyde, maintained at pH 7.4, and stored at 4 °C, whereas tissues prepared for RT-qPCR and western blots were snap frozen and stored at -80 °C for further analysis.

**Antibiotic treatment**

19-day-old (after weaning) KO mice were orally given for 3 consecutive days with 100 μL PBS (KO+PBS) or antibiotic cocktail that contained 1 g/L ampicillin, 1 g/L neomycin, 1 g/L metronidazole, and 0.5 g/L vancomycin hydrochloride (KO+Antibiotic). All the mice were singly housed after weaning with a plaything to allow for acclimation to the animal facility. Feces and tissues were collected when mice were 28 days old.

**High-fat diet feeding**

Three-week-old SIRT6-knockout (129Sv) and WT mice (129Sv) were fed a control standard AIN-93G diet (abbreviated as CD) containing 64% carbohydrates, 19% protein, and 17% fat or a high-fat diet (abbreviated as HD) consisting of AIN-93G with 65% of calories from fat, principally hydrogenated coconut oil (Table S2). The high-fat diet contained 16% carbohydrates, 19% protein, and 65% fat. The food supplied to the SIRT6 knockout mice should be accessible, and thus, we placed it, control or high-fat food, on the bedding considering the weakness and smaller body size of SIRT6 KO mice. In addition, the water bottle was also specially made to ensure the accessible of water for KO mice. The fiber and moisture content were the same in both control diet and high-fat diet, which were 5% (w/w) and 8% (w/w) respectively. The amounts of the high-fat and control diets were calculated as caloric intake per day per body weight with 4-week-old KO or WT mice. The dietary intervention lasted for 1 week. Feces collection and dissection were carried out after one week of high-fat diet feeding. For the calculation of survival rate, mice were raised to the end of the fifth week.

**Fecal microbiota transplantation**

3-month-old C57BL/6J male mice were used as recipients for microbiota transplantation. WT-WT (WT mice transplanted with fecal microbiota from WT mice), KO-WT (WT mice transplanted with fecal microbiota from SIRT6 KO mice), KOHD-WT (WT mice transplanted with fecal microbiota from KO mice who were fed with a high-fat diet). Before transplantation, mice were treated for 4 consecutive weeks with an antibiotic cocktail in drinking water that contained 1 g/L ampicillin (Sigma, MO, USA), 1 g/L neomycin (Sigma), 1 g/L metronidazole (Sigma), and 0.5 g/L vancomycin hydrochloride (BIORIGIN, Beijing, China). The drinking solution was renewed every 2 days. Then, mice were orally given 200 μL of the microbiota suspension every other day for 4 weeks (with each daily dose being administered by oral gavage after 2 h fast), starting the first day after the antibiotic cycle. Feces for further analysis were collected 72 hours after FMT. For the microbiota suspension preparation, 300 mg fecal pellets were resuspended with a vortex in 1 mL PBS, then centrifuged at 1,500 g for 5 min to remove insolubilized material and the supernatant was stored at -80 °C in a freezer. Feces collection and animal dissection were carried out after the microbiota transplantation. Body weight and tissue weight were recorded. Tissue samples were kept at 4 °C or -80 °C in a freezer, separately. Three to five fecal pellets of each mouse were randomly selected and the diameter of each fecal pellet was measured using vernier caliper. The average fecal diameter of each mouse was calculated and then presented in histogram.

FMT from WT or SIRT6 KO donor mice started when SIRT6 KO recipient mice were 19 days old (after weaning) without antibiotic treatment (Stebegg et al., 2019) (WT-KO: SIRT6 KO mice transplanted with fecal microbiota from WT mice; KO-KO: SIRT6 KO mice transplanted with fecal microbiota from SIRT6 KO mice). Feces from WT littermates or KO mice were collected just before the FMT and microbiota suspension was prepared as previously mentioned. Microbiota suspension was carefully administered orally to mice. SIRT6 KO mice were given microbiota suspension 200 μL per day for one week. After one-week period, mice received the microbiota suspension twice a week until natural death and the lifespan of mice was recorded. During FMT, cages of recipient mice (SIRT6 KO) were replenished with dirty bedding and fecal pellets from WT or KO mice three times a week. Understandably, the KO mice could obtain the gut microbiota from WT or KO as much as possible because of their coprophagy. Feces and tissues were collected when mice were 28 days old.

FMT from KOHD donor mice started when SIRT6 KO recipient were 19 days old (after weaning) without antibiotic treatment (KOHD-KO: SIRT6 KO mice transplanted with fecal microbiota from KOHD mice). Feces from KOHD mice were collected just before the FMT and microbiota suspension was prepared as previously mentioned. Microbiota suspension was carefully administered orally to mice. SIRT6 KO mice were given microbiota suspension 200 μL per day for one week. During FMT, cages of recipient mice (SIRT6 KO) were replenished with dirty bedding and fecal pellets from KOHD mice three times a week. Understandably, the KO mice could obtain the gut microbiota from KOHD as much as possible because of their coprophagy. Feces and tissues were collected when mice were 28 days old.

***E. coli* culture and oral supplementation**

Feces from SIRT6 knockout mice were resuspended in Tryptic Soy Broth (TSB) then incubated in 37 ℃ to mid-exponential phase. The cultures were centrifuged at 6,700 g for 5 min and the bacterial pellets were washed twice in phosphate-buffered saline (PBS). The bacterial suspension was then diluted in 100-fold serial dilutions and plated on *Escherichia coli* chromogenic medium (CHROM) (Solarbio, LA0780, Beijing, China). CHROM plates were incubated in the dark at 37 ℃ for a minimum of 24 h until the individual positive colony appeared. The positively stained colony was picked and seeded into 10 mL of LB medium (Solarbio, L1010, Beijing, China) for 37 ℃, 200 rpm incubation. When the CFU (Colony Forming Unit) of the LB medium reached 1.8 × 10^10^ CFU/mL, the bacteria were harvested by centrifuging under 9,000 g for 10 min. The bacterial pellets were washed twice in PBS and diluted to the concentration corresponding to 2 × 10^9^ CFU/kg body weight of each recipient WT mice. To ensure the enriched bacteria is Shiga toxin-producing *Escherichia coli* (STEC), about 100 mL bacterial suspension was boiled for 10 min and centrifuged at 13800g for 10 min to gain the genome DNA and qPCR assay was used to detect *Stx1*, *Stx2,* and *eaeA*.

STEC suspension (100 μL) were administrated by oral gavage to 3-month-old C57BL/6J male mice after 8 h of starvation for food. Control animals received 100 μL of sterile PBS. After 4 hours of ingestion of the bacterial suspension, both food and water were provided to the mice *ad libitum*. At 96 hours after infection, mice stools were collected to detect *E. coli*. After 1-month time, same STEC transplantation assay was operated. In another month, feces and organs were harvested for further analysis.

**Fecal water content assay**

Three to five fecal pellets of each mouse were randomly selected and the fecal water content of each fecal pellet was measured. The average fecal water content of each mouse was calculated and then presented in histograms. Fecal water content was measured as previously described (Jeong et al., 2017; Wang et al., 2020). Briefly, fecal pellets were collected into pre-weighed Petri dishes without lids, and the weight of each pellet was measured as the wet weight. Then the pellets were dried at 80 ℃ for 24 h, and the dry weight was measured. The water content of each fecal pellet was calculated according to the following equation:

$$fecal water content =\left( 1-\frac{fecal dry weight}{fecal wet weight} \right)\times100\%$$

**qPCR**

Total RNA was isolated from tissues after processing with TRIzol reagent (Invitrogen Life Technologies, NY, USA) following the manufacturer’s instructions. RNA concentrations and purity were estimated by determining the A260/A230 and A260/A280 ratio with a Thermo Scientific Nanodrop 2000c (Thermo, MA, USA). Reverse transcription of mRNA was performed using the cDNA Synthesis Kit (TIANGEN, Beijing, China). The kit contains gDNase which can efficiently remove genomic DNA, thus avoiding the interference of genomic DNA in Total RNA. PCR was carried out using SYBR Green (Yeasen, Shanghai, China) with CFX Manager 3.1 (Bio-Rad, CA, USA). Each sample was processed in triplicate and normalized to GAPDH or β-actin levels by the 2^−ΔΔCT^ method, and the values were expressed relative to those of the control group. Primers were ordered from Invitrogen and sequences were shown in Table S3.

For bacterial quantification, DNA from mouse feces was extracted using QIAamp Fast DNA Stool Mini Kit (QIAGEN, Hilden, Germany) according to the instructions. A 20 ng DNA sample was used for qPCR reactions using specific primers (Table S3) to amplify bacterial 16S rRNA. Bacterial abundance was normalized with the abundance of fecal total bacteria using the conserved eubacterial 16S rRNA primer pair. Results are presented as relative quantification.

For bacterial translocation analysis, spleen, kidney and liver tissues were weighted and genomic DNA was purified using TIANamp Genomic DNA Kit according to the manufacture’s protocol (TIANGEN, China). *E. coli* gene levels were determined by qPCR and normalized by the conserved eubacterial. Data were analyzed by relative quantification.

**Enzyme linked immunosorbent assay**

Mouse serum and fecal samples were assessed by ELISA according to the kit manufacturers’ instructions as follows: LPS, LCN2, IL-1β, TNFα and C-reactive protein (LCN2: Cloud Clone Corp., Wuhan, China; Others: CUSABIO, Wuhan, China). For the fecal sample, 40 mg of sample was first added to 0.2 mL PBS and pipetted thoroughly for 1 min; it was then balanced at room temperature (20-25 °C) for 10 min and finally centrifuged at room temperature at 6000-8000 RCF for 10 min. The supernatant was collected into a clean 1.5 mL tube for ELISA measurements. Serum samples were assessed based on the concentration and the instructions from the kit. Measurements were generally performed by adding 50 μL of supernatant and 50 μL detection antibody to each well, and samples were then tested according to the procedures stated in the manual from the kit. Raw data from standard curves and sample wells were optimized and analyzed using a GLOMAX Multi Detection System (Promega, WI, USA).

**16S rRNA sequencing analysis**

Microbial DNA was extracted from fecal samples and the 16S rRNA gene V4 region (515F-806R) of the isolated DNA with the barcode was amplified by PCR and sequenced using the Illumina HiSeq platform (service provided by Novogene Corporation, Beijing, China) following the manufacturer’s guidelines. Primer sequences were 515F: 5’-GTGCCAGCMGCCGCGGTAA, 806R: 5’-GGACTACHVHHHTWTCTAAT. All PCR reactions were carried out in 30 μL reactions with 15 µL of Phusion® High-Fidelity PCR Master Mix (New England Biolabs, MA, USA); 0.2 µM of forward and reverse primers, and about 10 ng template DNA. Thermal cycling consisted of initial denaturation at 98℃ for 1 min, followed by 30 cycles of denaturation at 98℃ for 10 s, annealing at 50℃ for 30 s, and elongation at 72℃ for 30 s, and finally at 72℃ for 5 min. USEARCH v10.0.240 was used for quality assurance and OTU picking for the raw sequences (Edgar, 2013).In brief, the raw sequences were first demultiplexed. Then, demultiplexed reads were merged into paired reads and the primer was stripped based on Vsearch (2.14.2). Merged reads with expected error thresholds larger than 1.0 or read lengths shorter than 160 were discarded as quality filtration. The Amplicon Sequence Variants non-clustering denoising was performed by Unoise3 of Usearch10. The quality filtered reads were dereplicated into unique sequences. Based on the abundances of the unique sequences, singletons were discarded. Then, the sequences were subjected to OTU clustering at 97% similarity. A chimera filter was built in this OTU clustering implementation based on the Silva database. After mapping all merged sequences picked by UPARSE to OTUs, a table was constructed. The OTU table was then subjected to QIIME 1.9.1 analysis (Kuczynski et al., 2011). The phylogenetic information of the OTUs was obtained using RDP classifier 11.5 with reference sequencing from 16S rRNA training set 16 of the Ribosomal Database Project using a bootstrap cutoff of 0.6 (Wang et al., 2007).

The gut microbiota diversity analysis and species taxonomy were based on alpha diversity, and beta diversity, shown as UniFrac distance displayed in the PCoA plot and LEfSe.

**Short chain fatty acid analysis**

Short chain fatty acid analysis was performed as described (Zhao et al., 2006). Fecal pellets from each mouse sample were weighted and approximately 100 mg was homogenized in 1 mL deionized water with 50% aqueous acetonitrile for 3 min using a stainless-steel bar. Next, the SCFAs were extracted by vortexing for 5 min. Then, the pH of the suspension was adjusted to 2-3 and the suspension was subsequently transferred to a polypropylene tube and centrifuged at 3,000 g for 20 min in 10 °C, after that the clear supernatant was collected. Chemical derivatization was performed by mixing 20 μL of 200 mM 3NPH in 50% aqueous acetonitrile and 20 mL of 120 mM EDC-6% pyridine solution with 40 μL of the supernatant after sample preparation. The mixture was reacted at 40 °C for 30 min and was then dried with a Speedvac™ Vacuum Concentrator (Thermo) and stored at -80 °C in a freezer. Fecal supernatant was spiked with standard solution in advance. Finally, distillates of the sample material were analyzed with the Exactive^TM^ GC Orbitrap^TM^ GC-MS system (Thermo) supported by the Metabolomics Core Facility Platform, Tsinghua University, and SCFAs including acetate, formate, propionate, isobutyrate, butyrate, isovalerate, and valerate, with > 50% above limit of detection (LOD) were assessed.

**Western blot**

To obtain proteins, the tissue mash was lysed in RIPA buffer (Biomiga, CA, USA) containing a protease inhibitor cocktail (AbMole Bioscience, Houston, USA) and phosphatase inhibitors (Solarbio, Beijing, China). The protein concentrations were measured using a BCA protein assay kit (Solarbio), following the instructions provided by the manufacturer. Then, proteins (50 μg/sample) were mixed with 6×loading buffer (Solarbio) and boiled for 5 min to denature them. Next, a 12% sodium dodecyl sulfate polyacrylamide gel was prepared in accordance with standard protocols. Standard electrophoresis was then performed and proteins were transferred onto polyvinylidene difluoride membranes (Millipore, MA, USA). The membrane was blocked with 5% skim milk and 0.1% fetal calf serum at 37 °C for 1 h. Then, it was incubated with primary antibodies against Sirt6 (1:1000, ab62739, Abcam, CA, UK), p16 (1:1000, ab51243, Abcam), p21 (1:1000, ab188224, Abcam), β-actin (1:1000, 4970S, Cell Signaling Technology) and GAPDH (1:1000, ab181602, Abcam) overnight. After incubation, the blot was washed three times with TBST and then incubated with TBST containing a 1:1000 dilution of horseradish peroxide-conjugated goat anti-rabbit antibody (Abcam) for 2 h at room temperature. After washing with TBST three times, the blot was developed with an ECL kit (Sigma) and visualized by Chemi Capture (CLINX, Shanghai, China). Images were taken and gray statistics were analyzed using Imaging Lab software.

**Histological staining**

All tissues were fixed in 4% paraformaldehyde (Servicebio, Wuhan, China) for 24 h at 4 °C and then embedded in paraffin. The samples were dehydrated, and 4 μm sections were studied. H&E staining, PAS staining, and Alcian blue staining were performed according to the manufacturer’s instructions (Servicebio). Photographs were captured using a light microscope (Axio Scan.Z1, Zeiss, Germany). Zen 2.3 (blue edition, Carl Zeiss Microscopy GmbH, 2011) was used for the morphological assessment based on a scoring system in a blinded manner as previously reported (Xu et al., 2020). Intestinal villi lengths were measured by using ImageJ (Fiji). Use “Analyze-Set Scale” to set the measuring scale, then use the straight-line tool to measure the intestinal villi lengths. Three fields of each section were randomly selected, and then all villi in this field were measured and the average villi length of each field were calculated. Average length of each field was presented in histograms.

The severity of inflammation (with a score ranging from 0 to 3, indicating no inflammation, mild, moderate, or severe), mucosal damage (with a score ranging from 0 to 3, indicating none, mucosa, submucosa, transmembrane), and crypt damage (with a score of 0 to 4, indicating none, one-third of basal are damaged, basal two-third are damaged, only epithelium is intact, and the entire crypt and epithelium are lost) were independently measured to access the histological score of colon and small intestine. Each parameter scored was multiplied by the percentage of tissue involved and the total was added up to obtain the histopathological score. Three fields of each section were randomly selected and scored. The average histological score of each section was calculated and then presented in histograms.

**Immunohistochemistry staining**

Immunohistochemistry (IHC) was performed on formalin-fixed, paraffin-embedded (FFPE) tissue. 5 μm paraffin sections were cut. After antigen retrieval with citrate solution, slides were rinsed and blocked with a peroxidase-blocking reagent and incubated with p16^INK4a^ antibody or p21^WAF1^ antibody (ab51243 and ab188224, Abcam). Immunoreactive signals were visualized with DAB Quanto chromogen (Servicebio). Then, slides were counterstained with hematoxylin, dehydrated, mounted, and covered with a coverslip. Immunoreactivity was visualized by a light microscope (Axio Scan.Z1). Images were analyzed using ImageJ (NIH, version:2.2.0/1.53c). p16 and p21 were quantified using a color deconvolution algorithm to identify DAB positivity in defined ImageJ-based macros regions of interest (ROI) for each field. Specific ROI was selected to exclude inappropriate regions. Those regions without nucleus-positive staining co-located were regarded as false positive regions. When calculating, these regions were excluded specifically. Percentages of positive cells were calculated for each field. Three fields of each section were randomly selected and measured. The average percentage of each section was calculated and then presented in histograms.

**SA-β-gal activity assay**

SA-β-gal staining was performed in accordance with the manufacturer’s instructions (Servicebio, G1073). Briefly, frozen tissues (8 μm thick) were rewarmed at room temperature for 10 min and then fixed in fix solution for 20 min at room temperature. The frozen sections were washed three times with PBS and then incubated with SA-β-gal staining solution (pH6.0) overnight at 37℃ without CO_2_. After completion of SA-β-gal staining, the sections were counterstained with eosin for 5 min and then rinsed with ddH_2_O for three times. Sections were dehydrated in absolute alcohol for two times and cleared in xylene for 5 min. Excess xylene was removed and a coverslip was placed over the section. After drying overnight at 4℃, the sample was observed and visualized by a light microscope (Axio Scan.Z1). Three fields of each section were randomly selected and measured. The average percentage of each section was calculated and then presented in histograms. Liver, spleen and kidney frozen sections stained with SA-β-gal were quantified by ImageJ software (NIH, version 2.1.0/1.53c) to measure the positive staining area. The total area was quantified by eosin-positive area. The relative SA-β-gal-positive area were calculated with the SA-β-gal-positive area divided by the total area. “Color threshold” was used to select target area, for SA-β-gal-positive area, the threshold was set as: Red (0,100), Green (0,20), Blue (0,200); for eosin-positive area, the threshold was set as: Red (100,255), Green (0,58), Blue (140,255). “Measure” restricted to threshold was used to measure the area. For the statistics of SA-β-gal-positive area of liver and spleen, the regions were randomly selected to be photographed. The SA-β-gal-positive area of kidney were randomly selected in the regions avoiding renal proximal tubular epithelium to avert false positives.

**Detection and discrimination of *E. coli* strain**

Fecal samples from SIRT6 KO mice (20 mg) were homogenized in 1mL of Trypto-casein-soy (TCS) broth (Solarbio). The fecal stock was diluted 100 times, 1,000 times and 10,000 times respectively using TCS broth and incubated at 37 ℃ for 3 h. 100 µL aliquots were plated on *E. coli* chromogenic media (Solarbio) and incubated at 37 ℃ for 20 h in aerobic conditions. *E. coli* chromogenic media is selective and differential medium for the isolation of all *Escherichia coli* and several non-fermenting Gram-negative bacteria. The overnight cultures were examined for bacterial growth and colony morphology. Aquamarine blue colonies on the chromogenic medium were *E. coli.* DNA was extracted from randomly selected aquamarine blue colonies and directly tested for the *aggR*, *eaeA*, *Stx1*, *Stx2*, *St (1a/1b)*, *It,* *ipaH*, and *daaD* genes by qPCR. Primer sequences were listed in Table S3.

**FITC-dextran intestinal permeability assay**

Gut permeability was measured using Fluorescein isothiocyanate (FITC)-dextran (average molecular weight: 4000 Da). Briefly, mice fasted for 6 h were intragastric administration with FITC-dextran (600 mg/kg body weight, 180 mg/mL). After 4 h, blood samples were collected and centrifuged (3000 g at 4 ℃) for 10 min, and serum were collected. GLOMAX Multi Detection System (Promega) was used to determine FITC-dextran concentration. Standard curves were produced by serial dilution of FITC-dextran in serum from untreated mice.

**Statistical analysis**

Results are expressed as means ± SEMs. Statistical significance was evaluated using two-tailed unpaired t test, one-way ANOVA or two-way ANOVA with Tukey’s multiple comparisons test. PERMENOVA tests were used for PCoA analysis. A *p*-value less than 0.05 was considered significant. Data were analyzed and plotted in Graph Pad Prism 9.0 software, STAMP software, or R version 3.6.2.

**References**

Edgar, R. C. (2013). UPARSE: highly accurate OTU sequences from microbial amplicon reads. *Nat Methods*, *10*(10), 996-998. <https://doi.org/10.1038/nmeth.2604>

Jeong, D., Kim, D. H., Kang, I. B., Kim, H., Song, K. Y., Kim, H. S., & Seo, K. H. (2017). Modulation of gut microbiota and increase in fecal water content in mice induced by administration of Lactobacillus kefiranofaciens DN1. *Food Funct*, *8*(2), 680-686. <https://doi.org/10.1039/c6fo01559j>

Kuczynski, J., Stombaugh, J., Walters, W. A., González, A., Caporaso, J. G., & Knight, R. (2011). Using QIIME to analyze 16S rRNA gene sequences from microbial communities. *Curr Protoc Bioinformatics*, *Chapter 10*, Unit 10.17. <https://doi.org/10.1002/0471250953.bi1007s36>

Stebegg, M., Silva-Cayetano, A., Innocentin, S., Jenkins, T. P., Cantacessi, C., Gilbert, C., & Linterman, M. A. (2019). Heterochronic faecal transplantation boosts gut germinal centres in aged mice. *Nature communications*, *10*(1), 1-13.

Wang, F., Huang, X., Chen, Y., Zhang, D., Chen, D., Chen, L., & Lin, J. (2020). Study on the Effect of Capsaicin on the Intestinal Flora through High-Throughput Sequencing. *ACS Omega*, *5*(2), 1246-1253. <https://doi.org/10.1021/acsomega.9b03798>

Wang, Q., Garrity, G. M., Tiedje, J. M., & Cole, J. R. (2007). Naive Bayesian classifier for rapid assignment of rRNA sequences into the new bacterial taxonomy. *Appl Environ Microbiol*, *73*(16), 5261-5267. <https://doi.org/10.1128/aem.00062-07>

Xu, K., Guo, Y., Ping, L., Qiu, Y., Liu, Q., Li, Z., & Wang, Z. (2020). Protective Effects of SIRT6 Overexpression against DSS-Induced Colitis in Mice. *Cells*, *9*(6). <https://doi.org/10.3390/cells9061513>

Zhao, G., Nyman, M., & Jönsson, J. A. (2006). Rapid determination of short-chain fatty acids in colonic contents and faeces of humans and rats by acidified water-extraction and direct-injection gas chromatography. *Biomedical Chromatography*, *20*(8), 674-682. <https://doi.org/10.1002/bmc.580>
